# Design and recognition of cucurbituril-secured platinum-bound oligopeptides†

**DOI:** 10.1039/d1sc02637b

**Published:** 2021-06-17

**Authors:** Héctor Barbero, Eric Masson

**Affiliations:** Department of Chemistry and Biochemistry, Ohio University Athens Ohio 45701 USA masson@ohio.edu

## Abstract

Platinum terpyridyl complexes, stacked on top of one another and secured as dimers with cucurbit[8]uril (CB[8]) in aqueous medium, were functionalized quantitatively and *in situ* with a pair of pentapeptides Phe-(Gly)_3_-Cys by grafting their cysteine residues to the Pt centers. The resulting CB[8]·(Pt·peptide)_2_ assemblies were used to target secondary hosts CB[7] and CB[8] *via* their pair of phenylalanine residues, again *in situ*. A series of well-defined architectures, including a supramolecular “pendant necklace” with hybrid head-to-head and head-to-tail arrangements inside CB[8], were obtained during the self-sorting process after combining only 3 or 4 simple building units.

## Abstract 2

A platinum terpyridyl complex, pentapeptide Phe-(Gly)_3_-Cys and cucurbit[8]uril assemble into a “pendant necklace” with hybrid head-to-head and head-to-tail arrangements in aqueous medium.

## Graphical abstract

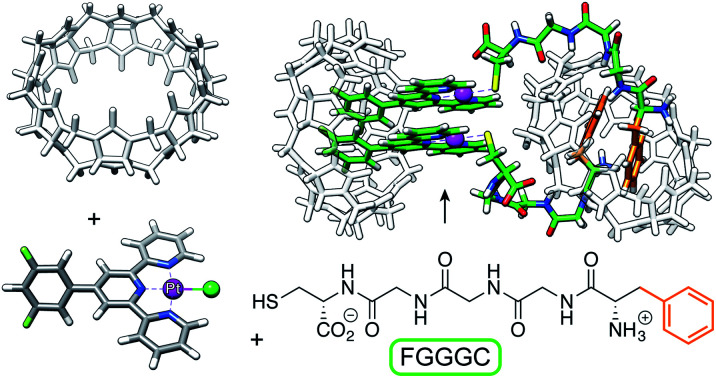

## Introduction

Cucurbit[8]uril (CB[8]), a member of the cucurbituril family of macrocycles,^1–5^ typically forms ternary complexes in aqueous medium with pairs of charged guests by distributing the positive charges over both portals of the macrocycle, in a head-to-tail (HT) arrangement. However, we recently showed that platinum terpyridyl (tpy) complexes bearing a CB[8]-binding unit at the tpy 4′-position assemble with CB[8] into a head-to-head (HH) motif. Both positive Pt centers sit on top of each other at one CB[8] portal, leaving the other void of any guest interaction.^6^ Favorable dispersive interactions between the stacked tpy ligands and possible metal–metal bonding through d_*z*^2^ −_ d_*z*^2^_ orbital overlap were proposed as driving forces for the recognition pattern. We showed later that a variety of thiolates, including cysteine and glutathione, can be grafted *in situ* to CB[8]-secured Pt chloride dimers to form large dynamic libraries of homo- and heteroternary assemblies.^7^

Grafting peptides onto the CB[8]-secured Pt dimer scaffold is a step towards two distinct longer term objectives: (1) the design of new well-defined motifs for protein recognition, and (2) the rational design of synthetic oligopeptides with specific or unusual conformations. The well-known cytotoxic properties of Pt complexes^8–13^ are also seen as an exploitable feature. In this proof-of-concept study, we wanted to test whether it was indeed possible to (1) form well-defined HH CB[8]-secured Pt/peptide dimers, and (2) use those assemblies to target a subsequent host, again in a well-defined manner. We show that both hypothesis are valid using pentapeptide Phe-(Gly)_3_-Cys (FGGGC) and either CB[7] or CB[8] as secondary hosts. We also show that elegant structures such as “pendant necklaces” with both HH and HT features can be obtained *in situ* from just a few readily available building blocks in aqueous medium. Pentapeptide FGGGC was chosen as terminal phenylalanines (Phe) form tight binary complexes with CB[7] (binding affinity up to 3.1 × 10^7^ M^−1^),^1,14,15^ and HT ternary assemblies with CB[8] (binding affinity up to 1.5 × 10^11^ M^−2^;^15,16^ see ESI† section for the titration of the pentapeptide with CB[8]). This feature was discovered by Urbach and coworkers,^15–23^ and was exploited by the same group to selectively encapsulate into CB[7] the N-terminal Phe residue of proteins, including the insulin B-chain,^24^ the human growth hormone (hGH),^25^ ubiquitin^26^ and myoglobin.^26^ Brunsveld and coworkers^27–33^ showed that protein dimerization is possible by exploiting the recognition ability of CB[8] towards pairs of Phe residues, leading to applications such as enzyme activity modulation.^29,31^ Liu also showed that the same motif can be used to engineer large protein-based nanostructures.^34–37^ In our system, the C-terminal cysteine residue of FGGGC binds to the Pt centers, and the three glycines act as spacers that confer flexibility to the side chain. For the sake of clarity, we will add “Pt” or “Phe” superscripts to CB[*n*] to indicate which portion of the Pt/peptide complex the macrocycles interact with.

## Results and discussion

**Fig. 1:**
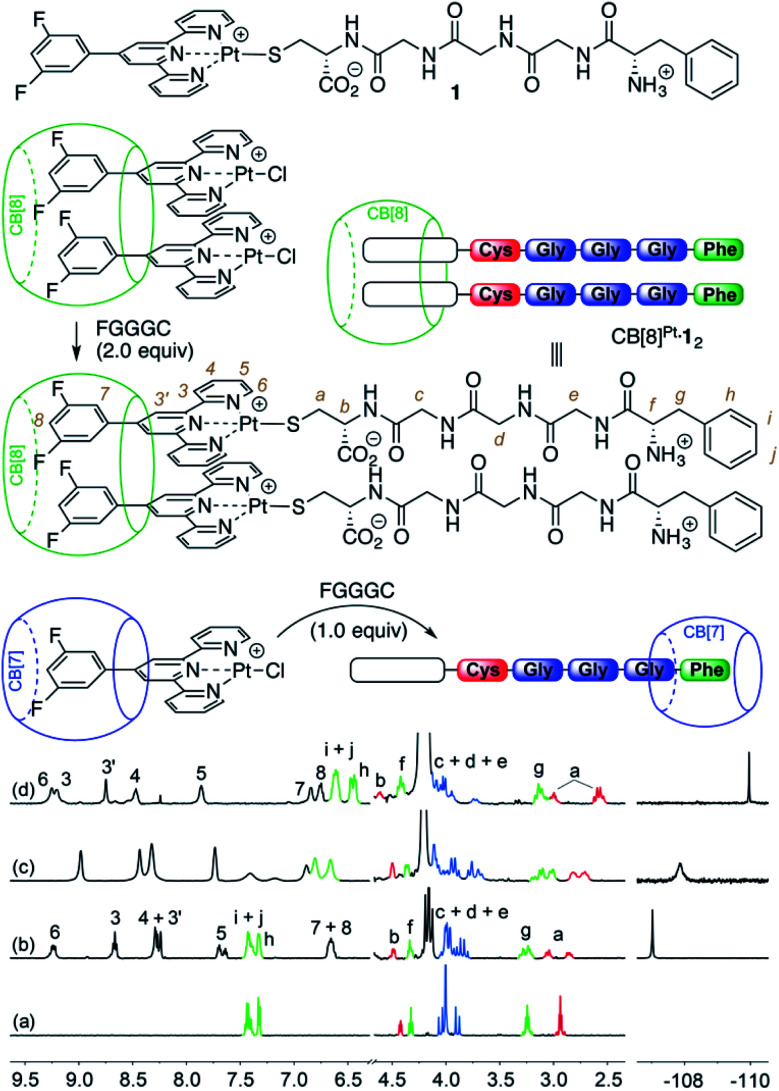
Formation of CB[*n*]-bound Pt/peptide assemblies. ^1^H and ^19^F NMR spectra of (a) free peptide FGGGC, as well as assemblies (b) CB[8]^Pt^·**1**_2_, (c) **1**·CB[7]^Phe^ and (d) CB[7]^Pt^·**1**·CB[7]^Phe^ in D_2_O at 25 °C. Signals pertaining to the amino acids are colored according to the cartoon representation. All chemical shifts in this figure and in the following ones are in ppm.

Pt/peptide assembly CB[8]^Pt^·**1**_2_ (see Fig. 1) was readily obtained *in situ* by substitution of the chloride ligand from the parent Pt chloride assembly with pentapeptide FGGGC in deuterium oxide. No buffer was used as the charge of Pt/peptide complex **1** is expected to remain singly positive on a wide pH (or pD) range (approximately 2–9). Upon dimer formation at the Pt tpy site, terpyridyl hydrogens H^6^ are shifted downfield by 0.33 ppm and split into two doublets, as they become diastereotopic in the presence of the two chiral peptides (see Fig. 1, spectrum b). This behavior was also observed for aromatic hydrogens at positions 3–5 and 3′, as expected from one of our earlier studies.^7^ Signals pertaining to peptide FGGGC are barely affected except for diastereotopic hydrogens H^*a*^, which split into two multiplets separated by 0.08 ppm. On the other hand, fluorine atoms located at the tpy head experience an upfield shift (0.19 ppm) upon peptide binding.

Treatment of the CB[7]-bound Pt chloride assembly with the pentapeptide, however, afforded mostly assembly **1**·CB[7]^Phe^, with CB[7] switching from the Pt tpy to the Phe station (see Fig. 1, spectrum c). In the presence of an excess amount of CB[7] (>2 equiv.), both units were encapsulated by the macrocycle (see Fig. 1, spectrum d).

**Fig. 2:**
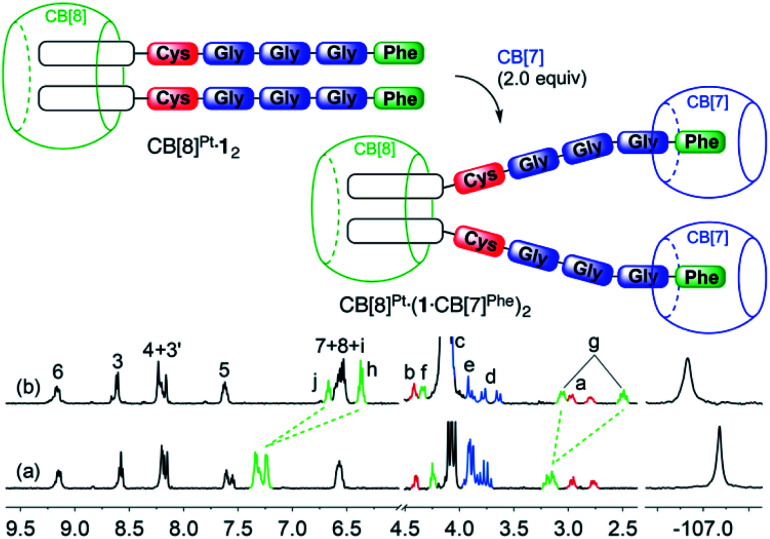
Recognition of CB[7] by assembly CB[8]^Pt^·**1**_2_. ^1^H and ^19^F NMR spectra of assemblies (a) CB[8]^Pt^·**1**_2_, and (b) CB[8]^Pt^·(**1**·CB[7])_2_ in D_2_O at 25 °C; see Fig. 1 for hydrogen labeling. Ticks 0.1 ppm apart on the ^19^F NMR spectra chemical shift axis.

We then titrated assembly CB[8]^Pt^·**1**_2_ with CB[7] to test the stability of the CB[8]-secured Pt dimer in the presence of a competing target host. [5]Pseudorotaxane CB[8]^Pt^·(**1**·CB[7]^Phe^)_2_ was formed exclusively after addition of 2.0 equiv. CB[7] (see Fig. 2). No association between the tpy head and CB[7] was observed, *i.e.* no disassembly of the CB[8]-secured dimer took place. Significant upfield shifts were observed for aromatic hydrogens H^*h*^, H^*i*^ and H^*j*^ (0.64–0.88 ppm) and methylene hydrogens H^*g*^ (0.64 ppm), and downfield shifts for hydrogens H^*f*^ (0.10 ppm). Those shifts confirm the inclusion of the benzyl moiety within CB[7], with the α-carbonyl hydrogen H^*f*^ located near the rim of the macrocycle. As expected, no significant shifts were observed for the remote fluorine nuclei on the tpy heads.

**Fig. 3:**
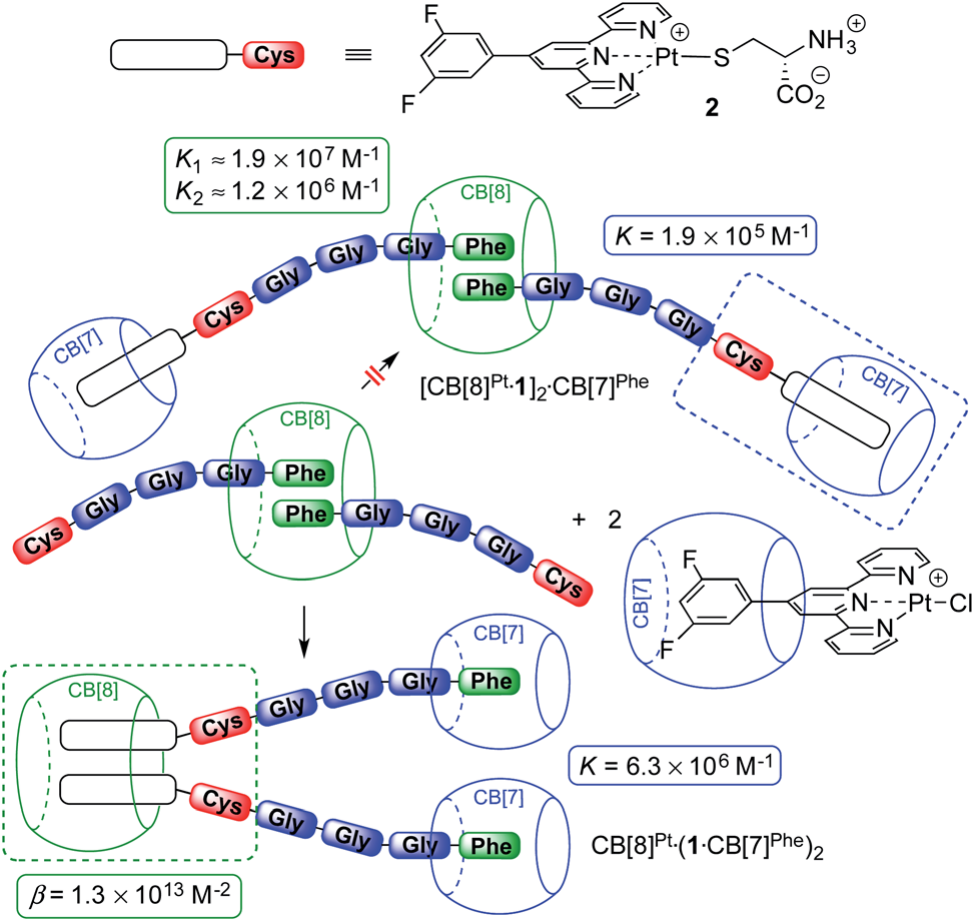
CB[7] and CB[8] switching stations upon addition of CB[8]-bound peptide dimer (FGGGC)_2_·CB[8] to the CB[7]-bound Pt chloride precursor. Binding affinities obtained by isothermal titration calorimetry.

Remarkably, [5]pseudorotaxane CB[8]^Pt^·(**1**·CB[7]^Phe^)_2_ is also formed exclusively when ternary assembly (FGGGC)_2_·CB[8] is treated with 2.0 equiv. of the CB[7]-bound Pt tpy chloride precursor (see Fig. 3). The expected [CB[7]^Pt^·**1**]_2_·CB[8]^Phe^ assembly was not detected, even immediately after mixing.

To quantify the CB[8] preference for the Pt tpy sites over the Phe residues, the various recognition events at play were analyzed by isothermal titration calorimetry (ITC) in pure water.

The affinities of CB[7] towards the Phe residue of pentapeptide FGGGC and the CB[8]^Pt^·**1**_2_ complex are 1.2 (±0.2) × 10^7^ M^−1^ and 6.3 (±0.2) × 10^6^ M^−1^ (see Fig. 3), respectively, in excellent agreement with the binding affinities measured by Urbach for the FGG peptide (3.1 × 10^7^ M^−1^ in a 10 mM sodium phosphate buffer, pH 7.0).^15,20^ The proximity of both peptide chains in complex CB[8]^Pt^·**1**_2_ thus does not cause any cooperativity effect, *i.e.* grafting the peptide to the CB[8]-secured Pt dimer scaffold does not significantly impact the binding affinity of the terminal Phe unit. To determine the affinity of CB[7] towards the difluoroaryl substituent of the tpy ligand without perturbation from the Phe binding site, titrations were carried out using truncated Pt/cysteine complex **2** highlighted with a dashed blue box in Fig. 3. The affinity of this complex towards CB[7] is only 1.9 (±0.1) × 10^5^ M^−1^. The affinities of pentapeptide FGGGC towards CB[8] were 1.9 (±0.6) × 10^7^ M^−1^ and 1.2 (±0.6) × 10^6^ M^−1^ for the formation of the binary and HT ternary complexes, respectively, again in excellent agreement with reported binding affinities of N-terminal Phe residues in short peptides^15,18,38–40^ and proteins.^29,31,34^ Cooperativity is quantified using eqn (1), where *K*_1_ and *K*_2_ are the equilibrium constants for the formation of binary and ternary complexes, and *α* is an interaction parameter; positive and negative cooperativities are observed when *α* > 1 and *α* < 1, respectively.^41,42^ In our case, cooperativity in the CB[8] encapsulation of the pair of Phe residues is slightly negative (*α* = 0.26 ± 0.15).1
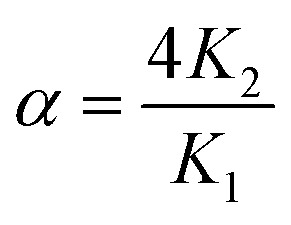


A word of caution is warranted, however. As shown by Urbach and coworkers in the case of tripeptide FGG and CB[8],^18^ as well as Cistola and coworkers with small molecule/protein interactions,^43^*K*_1_ and *K*_2_ constants obtained by ITC can be strongly correlated, *i.e.* (1) reasonable fits of ITC enthalpograms can be obtained when setting *K*_1_ as a constant while fitting *K*_2_; and (2) ternary binding constant *β* = *K*_1_*K*_2_ (in M^−2^) is rather insensitive to the value of *K*_1_. In our case, a plot of the goodness-of-fit value *χ*^2^ as a function of *K*_1_ returns a clear minimum at the *K*_1_ constant mentioned above (see ESI section, Fig. S33a†). Furthermore, the error on parameter *α* is small enough to ascertain that negative cooperativity is much more likely than not.

We used again truncated Pt/cysteine complex **2** to determine the affinity of CB[8] towards the difluoroaryl unit of the tpy ligand (highlighted with a dashed green box in Fig. 3). Equilibria (2) and (3) were used to fit the enthalpogram (see ESI section, Fig. S32f†), returning equilibrium constants *K*_Pt–Pt_ and *β* equal to 2.0 (±1.1) × 10^4^ M^−1^ and 1.3 (±0.6) × 10^13^ M^−2^, respectively.2
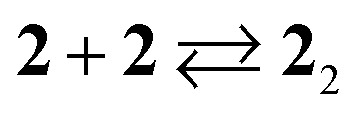
3
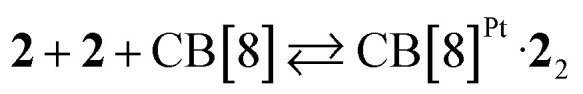


The dimerization constant *K*_Pt–Pt_ of assembly **2** corresponds to a free energy term of −5.9 (±0.3) kcal mol^−1^, in excellent agreement with the typical strength of Pt–Pt interactions.^44^ We note that this dimerization in the absence of CB[8] prevents us from extracting separate binding constants *K*_1_ and *K*_2_ towards the macrocycle. A reliable ternary binding constant *β* = *K*_1_*K*_2_ was obtained, however (1.3 (±0.6) × 10^13^ M^−2^). In other terms, dimer **2**_2_ can be considered as a standalone guest forming a 1 : 1 complex with CB[8], with a binding affinity *K*′ of 7 (±5) × 10^8^ M^−1^, obtained from eqn (4).4
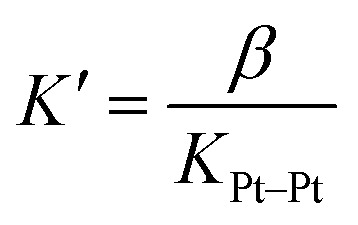


The combined equilibrium constant for the formation of assemblies [CB[7]^Pt^·**1**]_2_·CB[8]^Phe^ (upper section of Fig. 3, not observed) and CB[8]^Pt^·(**1**·CB[7]^Phe^)_2_ (lower section of Fig. 3) from the free hosts and guests are thus 8.1 × 10^23^ and 5.2 × 10^26^ M^−4^, respectively. This 640-fold difference corresponds to a 3.8 kcal mol^−1^ preference for assembly CB[8]^Pt^·(**1**·CB[7]^Phe^)_2_, which supports its exclusive formation.

**Fig. 4:**
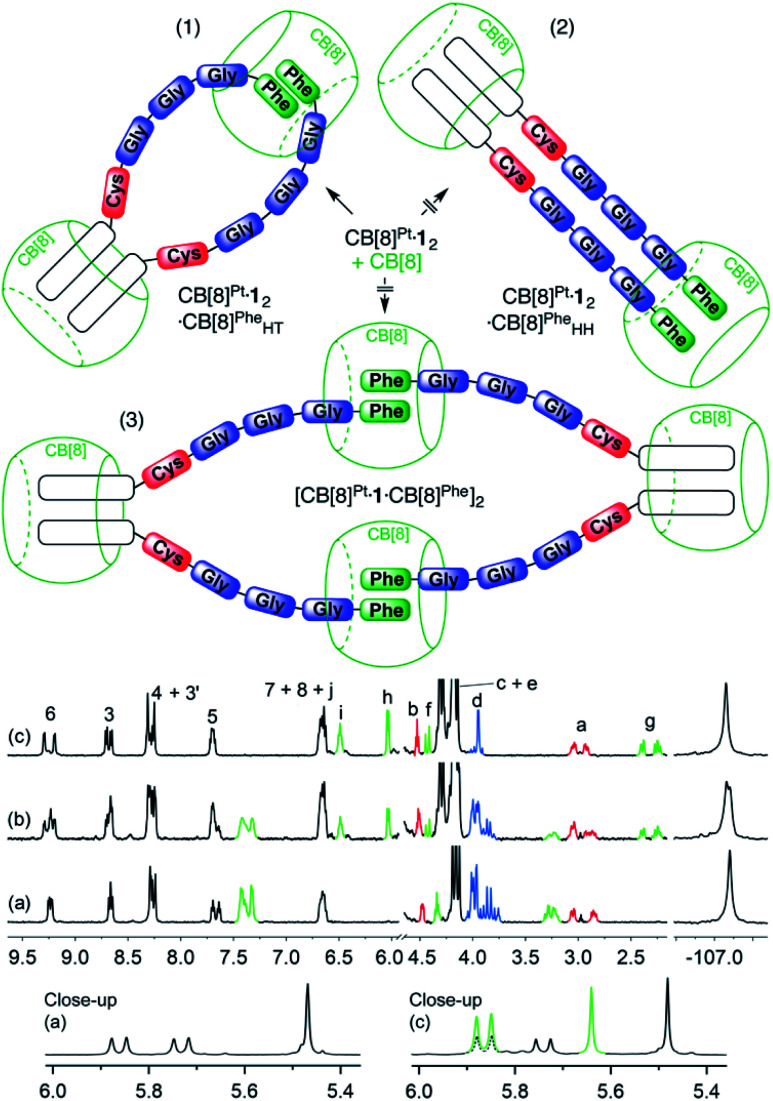
Three scenarios (1–3) considered for the recognition of CB[8] by Pt dimer CB[8]^Pt^·**1**_2_. ^1^H and ^19^F NMR spectra of assembly CB[8]^Pt^·**1**_2_ in D_2_O at 25 °C (a) in the absence of CB[8], and in the presence of (b) 0.50 equiv. and (c) 1.0 equiv. CB[8] relative to assembly CB[8]^Pt^·**1**_2_; see Fig. 1 for signal labeling. Close-up of spectra (a) and (c) in the 5.3–6.0 ppm region. Signals pertaining to CB[8]^Phe^ are highlighted in green; dashed signals highlight the signal overlap with CB[8]^Pt^ hydrogens. Ticks 0.1 ppm apart on the ^19^F NMR spectra chemical shift axis.

We subsequently tested the recognition of CB[8]-secured Pt dimer CB[8]^Pt^·**1**_2_ towards CB[8] as the target host. In the presence of 0.5 equiv. of the macrocycle (relative to Pt), *i.e.* 1.0 equiv. relative to assembly CB[8]^Pt^·**1**_2_, at least three well-defined assemblies are plausible (see Fig. 4): (1) a supramolecular “pendant necklace” with a 2 : 2 Pt-peptide/CB[8] stoichiometry in hybrid HH and HT arrangements at the tpy units and Phe residues, respectively (CB[8]^Pt^·**1**_2_·CB[8]^Phe^_HT_), (2) a structure with the same stoichiometry but in a double HH arrangement (CB[8]^Pt^·**1**_2_·CB[8]^Phe^_HH_), and (3) a HT dimer of assemblies with a 4 : 4 stoichiometry [CB[8]^Pt^·**1**_2_·CB[8]^Phe^]_2_.

New ^1^H NMR signals appeared immediately after adding a first CB[8] aliquot (0.50 equiv.; see Fig. 4, spectrum b), showing the formation of a new assembly in a slow exchange regime. A very well-defined set of new signals is present after addition of exactly 1.0 equiv. CB[8] relative to assembly CB[8]^Pt^·**1**_2_ (spectrum c). This led us to suspect that only one of the assemblies outlined above is present in the mixture. The formation of a new, unique ^19^F NMR signal at 1.0 equiv. CB[8] (−107.06 ppm) is consistent with the observations made from the ^1^H NMR spectra.

A comparison of spectra a and c in Fig. 4 shows a large upfield shift for hydrogens H^*g*^, H^*h*^, H^*i*^ and H^*j*^ (0.90, 1.29, 0.79 and 0.90 ppm, respectively), a similar behavior already observed for aromatic hydrogens in the CB[8]-bound peptide dimer FGGGC_2_·CB[8] (see ESI section, Fig. S31†). This clearly indicates that the recognition event is taking place at the Phe residue. The 5.3–6.0 ppm portion of the ^1^H NMR spectrum displays a feature that also allows us to discard assembly CB[8]^Pt^·**1**_2_·CB[8]^Phe^_HH_ with the double HH arrangement (see Fig. 4, scenario 2): the CB[8] rims in such an assembly would all be non-equivalent, and would afford 2 pairs of doublets for the pseudo-equatorial hydrogens. This is not the case; addition of 0.50 equiv. CB[8] to assembly CB[8]^Pt^·**1**_2_ shows the growth of only one doublet at 5.85 ppm (see Fig. 4, green signal in spectrum c close-up), in addition to the expected signal of the new CB[8]^Phe^ equatorial hydrogens (5.65 ppm, also in green). This is evidence that the newly bound CB[8]^Phe^ has equivalent portals. In order to discriminate between assembly CB[8]^Pt^·**1**_2_·CB[8]^Phe^_HT_ and dimer [CB[8]^Pt^·**1**_2_·CB[8]^Phe^]_2_ (see Fig. 4, scenarios 1 and 3), we compared the diffusion coefficients of the unknown assembly with that of complex CB[8]^Pt^·**1**_2_ using diffusion-ordered spectroscopy (DOSY) experiments.^40,45–53^ Diffusion constants in D_2_O were 1.51 (±0.01) × 10^−10^ m^2^ s^−1^ and 1.58 (±0.04) × 10^−10^ m^2^ s^−1^, respectively. A difference as low as 7 (±5) × 10^−12^ m^2^ s^−1^ indicates that the hydrodynamic radius of the unknown assembly is very similar to CB[8]^Pt^·**1**_2_ (within 0.7 (±0.4) Å).^40^ Assembly CB[8]^Pt^·**1**_2_·CB[8]^Phe^_HT_, with hybrid HH and HT arrangements at the tpy ligand and Phe residues, respectively (see Fig. 4, scenario 1) thus becomes the only plausible option. It is preferred over the entropically penalizing 4 : 4 assembly [CB[8]^Pt^·**1**_2_·CB[8]^Phe^]_2_. Furthermore, in two earlier studies,^47,50^ we showed that the molecular weights *M* of metal tpy complexes that self-assemble into rigid dynamic oligomers in the presence of CB[8] are linked to their diffusion coefficients *D* by the empirical power law (5), with *m* = 0.41 (±0.03) and *b* = 8.28 (±0.09). A coefficient *m* close to 1/3 would characterize spherical particles according to the Stokes–Einstein equation, while *m* usually ranges from 0.3 to 0.6 in non-spherical systems like oligomers.^54^

5 −log *D* = *m* log *M* + *b*

Power law (5) returns *M* = 5200 (±600) g mol^−1^ for putative assembly CB[8]^Pt^·**1**_2_·CB[8]^Phe^_HT_, in remarkable agreement with its actual molecular weight (4617 g mol^−1^). Finally, mass spectrometry analysis also confirmed the formation of this “pendant necklace” (see ESI section†).

Binding constants for the formation of the binary and ternary complexes at the Phe residue were *K*_1_ = 2.4 (±0.3) × 10^6^ M^−1^ and *K*_2_ = 1.7 (±0.4) × 10^6^ M^−1^, respectively. Again, a plot of the goodness-of-fit value *χ*^2^ as a function of *K*_1_ returns a clear minimum at the *K*_1_ constant mentioned above (see ESI section, Fig. S33b†). While attempts to rationalize the mild differences with the CB[8] encapsulation of free pentapeptide FGGGC would be putative, two trends deserve mentioning: (1) positive cooperativity is now observed (*α* = 2.8 ± 0.7) while cooperativity is negative in the case of free peptide FGGGC (*α* = 0.26 ± 0.15); and (2) the formation of the “pendant necklace” (*i.e.* the formation of the ternary complex at the Phe residues) is entropically neutral (*T*Δ*S*_2_ = −0.1 kcal mol^−1^), while ternary complex formation with the free peptide is entropically penalizing (*T*Δ*S*_2_ = −2.0 kcal mol^−1^). Both trends suggest that the entropically favorable “intra-assembly” necklace formation overcompensates the concomitant, entropically-penalizing restriction of conformational mobility.

We note here that we use the term “pendant necklace” for the lack of a better word to qualify structure CB[8]^Pt^·**1**_2_·CB[8]^Phe^_HT_. Topologically, it is a [2]pseudocatenane, with CB[8]^Phe^ being one ring component and the chain **1**·CB[8]^Pt^·**1** the other ring component. However, this simple nomenclature would consider the chain **1**·CB[8]^Pt^·**1** as one unit, without taking into account that it is itself a [3]pseudorotaxane.^55,56^ Ultimately, we propose the term “pendant necklace”, as the CB[8]-secured Pt tpy dimer reminds us of the pendant unit of the jewelry, and the HT ternary complex between the pair of Phe residues and CB[8]^Phe^ its clasp behind the neck. To the best of our knowledge, based in part on the recent review by Wang, Kermagoret and Bardelang on oligomeric CB[8] complexes,^57^ the formation of this hybrid HH/HT necklace is unprecedented.

**Fig. 5:**
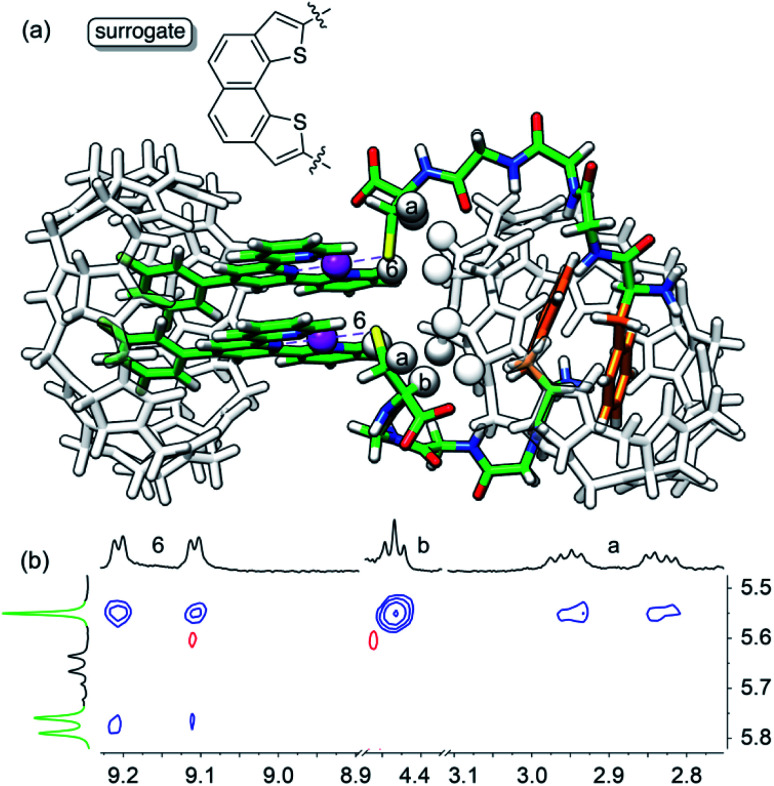
(a) Most stable structure of pendant necklace CB[8]^Pt^·**1**_2_·CB[8]^Phe^_HT_ optimized at the B97-3c/def2-mTZVP level. Hydrogen atoms interacting through space are highlighted with white spheres. (b) ^1^H–^1^H NOESY spectrum in D_2_O at 25 °C of assembly CB[8]^Pt^·**1**_2_·CB[8]^Phe^_HT_ where cross-peaks of hydrogens H6, H^*a*^ and H^*b*^ with CB[8]^Phe^ hydrogens are observed; signals pertaining to CB[8]^Phe^ appear in green.

As obtaining crystals of the pendant necklace for X-ray diffraction analysis was unsuccessful, we explored its structure using the most up-to-date combination of molecular dynamics, semi-empirical methods and density functional theory (DFT) being currently developed by Grimme and co-workers. Conformational screening was first carried out using an approximate geometrical analog of the pendant necklace bearing only one CB[8] unit (see Fig. 5a and S34†) with Grimme's Conformer–Rotamer Ensemble Sampling Tool (CREST)^58,59^ and the built-in generic GFN-Force-Field (GFN-FF),^60^ in conjunction with the GBSA solvation model.^61,62^ The CB[8]-secured Pt dimer surrogate was designed to mimic the distance between the sulfur atoms and the direction of its substituent in the pendant necklace, while increasing computing efficacy. The 37 most stable candidates (out of approximately 12 000; a 25 kcal mol^−1^ cut-off was applied) were isolated, and the surrogate fragment was replaced by the CB[8]-secured Pt dimer motif. The structures were then reoptimized with the semiempirical tight-binding method GFN2-xTB^63,64^ in conjunction with the GBSA solvation model.^61,62^ The four most stable structures (based on a 10 kcal mol^−1^ cut-off) were finally reoptimized by DFT using a functional well suited for supramolecular systems (B97-3c;^65^ basis sets are def2-mTZVP)^66,67^ in conjunction with the COSMO solvation model.^68,69^ The relative stabilities (Δ*G*) of the four assemblies were calculated using eqn (6), where Δ*E*_B97-3c_ is the electronic contribution at 0 K calculated by DFT in the gas phase, Δ*G*_T,xTB_ is the vibrational contribution at 25 °C obtained at the GFN2-xTB level, and Δ*G*_solv,xTB_ is the solvation energy calculated with the GBSA model at the GFN2-xTB level.

6 Δ*G* = Δ*E*_B97-3c_ + Δ*G*_T,xTB_ + Δ*G*_solv,xTB_

The most stable conformation of the pendant necklace is presented in Fig. 5. Its compact arrangement suggests that through-space interactions between hydrogens of the peptide and of CB[8] might be observable. A ^1^H–^1^H NOESY experiment indeed revealed cross-peaks between equatorial hydrogens of CB[8]^Phe^ and tpy hydrogen H^6^, as well as peptidic hydrogens H^*a*^ and H^*b*^ (see Fig. 5). Favorable interactions between the peptide carboxylate units and the outer wall of CB[8]^Phe^ might also further compact the assembly.^50^

## Conclusions

We showed that we could functionalize CB[8]-secured Pt dimers *in situ* and quantitatively with a pair of cysteine-containing peptides, and use the Pt/peptide/CB[8] assembly to target secondary hosts CB[7] and CB[8] site-selectively. The most elegant outcomes of the study are (1) the formation of pendant necklace CB[8]^Pt^·**1**_2_·CB[8]^Phe^_HT_ with a new hybrid HH and HT arrangement at the tpy head and Phe residues, respectively, and a thorough quantification of all recognition forces at play, (2) the successful *in silico* screening and isolation of a plausible geometry for this pendant necklace, and (3) the quantitative switching of CB[7] and CB[8] from the tpy head and Phe residues, respectively, when attempting to attach the Cys units of ternary complex **1**_2_·CB[8] to the Pt centers of binary assembly CB[7]^Pt^·(Pt·Cl). This study now paves the way for the recognition of proteins by these CB[8]-secured Pt dimers, and for the design of rationally constrained oligopeptides.

## Data availability

All analytical data is provided in the ESI. All details about computational methods and associated coordinates are available in the ESI.

## Author contributions

EM and HB conceived the project. HB conducted all experiments. EM and HB wrote the manuscript.

## Conflicts of interest

There are no conflicts to declare.

## Supplementary Material

SC-012-D1SC02637B-s001
