# Supplementary material for: Design and recognition of cucurbituril-secured platinum-bound oligopeptides
Source: Chem Sci. 2021 Jun 17;12(29):9962–8. doi: 10.1039/d1sc02637b (PMC8317623; doi:10.1039/d1sc02637b)
Supplement: SC-012-D1SC02637B-s001 [file SC-012-D1SC02637B-s001.pdf]

Electronic Supplementary Material (ESI) for Chemical Science.
This journal is © The Royal Society of Chemistry 2021

Design and Recognition of Cucurbituril-secured Platinum-bound Oligopeptides

Héctor Barbero and Eric Masson*

Department of Chemistry and Biochemistry, Ohio University, Athens, Ohio 45701, United States

e-mail: masson@ohio.edu

Supporting Information

1. Generalities 2
2. Preparation and characterization of CB[n]-bound Pt(tpy) chloride complexes 2
3. Preparation and characterization of Pt/peptide/CB[n] assemblies 3
4. ^{1}H and ^{13}C NMR spectra of the Pt/peptide/CB[n] assemblies 6
5. DOSY analysis for assemblies CB[8]^{Pt}·$\mathbf{1}_2$ and CB[8]^{Pt}·$\mathbf{1}_2$·CB[8]$^{Phe}{}_{HT}$ 16
6. Mass spectrometry analysis of Pt/peptide/CB[n] assemblies 19
7. UV-Vis absorption spectra of the Pt/peptide/CB[n] assemblies 22
8. Titration of peptide FGGGC with CB[8] 22
9. Isothermal Titration Calorimetry (ITC) experiments 23
10. Computational details 25
11. Coordinates of the most stable structure of pendant necklace CB[8]^{Pt}·$\mathbf{1}_2$·CB[8]$^{Phe}{}_{HT}$ 26
12. References 28

1. Generalities

All reagents were purchased from chemical suppliers and used without further purification. Peptide FGGGC (98% purity) was purchased from KareBay Biochem, Inc., Monmouth Junction, NJ. Cucurbit[7]uril (CB[7]) and Cucurbit[8]uril (CB[8]) were prepared using known procedures.[1] Solvents were of analytical grade and either used as purchased or dried according to procedures described elsewhere.[2] Characterization by Nuclear Magnetic Resonance spectroscopy (NMR) was carried out using a Bruker Ascend 500 MHz spectrometer and a Bruker Avance III HD Ascend 700 MHz located in the Campus Chemical Instrument Center (CCIC) NMR facility at The Ohio State University (OSU). ^{1}H and ^{13}C NMR chemical shifts are reported in parts per million (ppm) and are referenced to TMS using the residual signal of the solvent as an internal reference. Coupling constants (J) are reported in hertz (Hz). Standard abbreviations used to indicate multiplicity are: s = singlet, d = doublet, t = triplet, q = quartet, m = multiplet. Ultrahigh resolution/accurate mass measurements were carried out on a Bruker SolariXR 15T Fourier transform ion cyclotron resonance (FT-ICR) instrument. Positively charged ions were generated by electrospray ionization (ESI) and matrix assisted laser desorption/ionization (MALDI; α-cyano hydroxycinnamic acid was used as matrix). ESI produced multiply charged ions that were used to confirm product identity. ESI solutions (approx. 5 μM) in acetonitrile/water 1:1 were sprayed by direct infusion. The resolution of the FT-ICR instrument was set to 300,000 (at m/z 400). For MALDI, a Yag-Nd laser was used (351 nm; 15% of total power). UV-Vis absorption spectra were recorded on an Agilent HP-8453 diode-array spectrophotometer. Wavelengths (λ) are reported in nanometers (nm) and molar absorption coefficients (ε) are reported in $M^{-1}\cdot cm^{-1}$. Computational work was carried out on the Owens cluster of the Ohio Supercomputer Center in Columbus, OH (23,392-core Dell Intel Xeon E5-2680 v4 machines). 4'-(3,5-Difluorophenyl)-2,2':6',2''-terpyridine and chloro[4'-(3,5-difluorophenyl)-2,2':6',2''-terpyridine]-platinum(II) chloride (Pt·Cl) were prepared according to published procedures.[3]

2. Preparation and characterization of CB[n]-bound Pt(tpy) chloride complexes

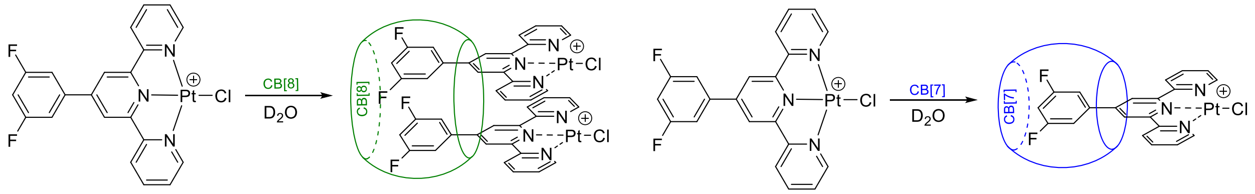

Chloro[4'-(3,5-difluorophenyl)-2,2':6',2''-terpyridine]platinum(II) chloride (3.1 mg, 5.1 μmol) was mixed with D_2O (5.0 mL). CB[8] (3.3 mg, 2.5 μmol) or CB[7] (5.9 mg, 5.1 μmol) was added subsequently, and the resulting mixture was sonicated thoroughly. The stock solution was stored at 4 ºC for further use.

CB[8]^{Pt}·(Pt·Cl)$_2$. ^{1}H NMR (500 MHz, D_2O) δ 8.81 (dd, J = 5.7, 1.5 Hz, 4H, H^6), 8.60 (d, J = 8.0 Hz, 4H, H^3), 8.19 (td, J = 7.8, 1.5 Hz, 4H, H^4), 8.16 (s, 4H, $H^{3'}$), 7.59 (ddd, J = 7.4, 5.6, 1.3 Hz, 4H, H^5), 6.64 – 6.52 (m, 6H, H^7+H^8), 5.78 (d, J = 15.3 Hz, 8H, $H^{CB[8]}$), 5.66 (d, J = 15.3 Hz, 8H, $H^{CB[8]}$), 5.40 (s, 16H, $H^{CB[8]}$), 4.11 (d, J = 10.5 Hz, 8H, $H^{CB[8]}$), 4.08 (d, J = 10.5 Hz, 8H, $H^{CB[8]}$). ^{19}F NMR (471 MHz, D_2O) δ -106.84.

CB[7]^{Pt}·(Pt·Cl). ^{1}H NMR (500 MHz, D_2O) δ 9.11 (d, J = 8.2 Hz, 4H, H^6), 8.90 (d, J = 5.8 Hz, 4H, H^3), 8.59 (s, 4H, H^4), 8.36 (t, J = 7.9 Hz, 4H, $H^{3'}$), 7.76 (t, J = 6.7 Hz, 4H, H^5), 6.76 (d, J = 7.3 Hz, 4H, H^7), 6.53 (t, J = 8.8 Hz, 2H, H^8), 5.73 (d, J = 15.4 Hz, 8H, $H^{CB[7]}$), 5.63 (d, J = 15.3 Hz, 8H, $H^{CB[7]}$), 5.39 (s, 16H, $H^{CB[7]}$), 4.10 (t, J = 15.8 Hz, 16H, $H^{CB[7]}$).

3. Preparation and characterization of Pt/peptide/CB[n] assemblies

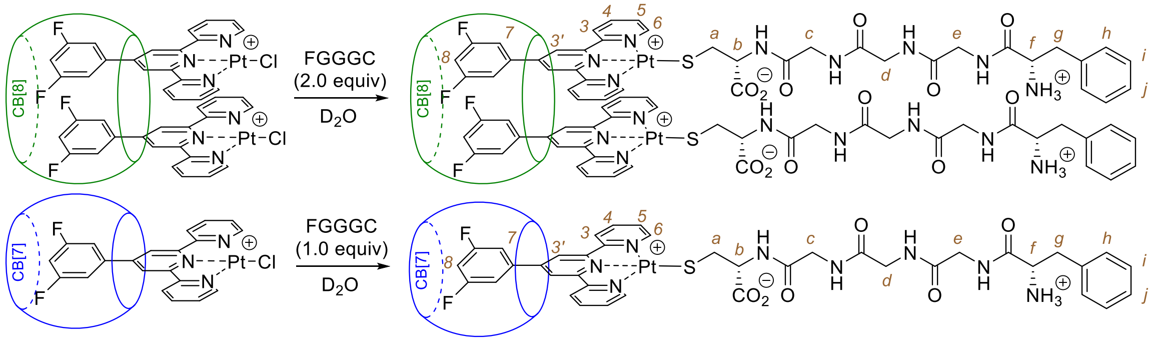

In an NMR tube, stock solutions of complexes CB[8]^{Pt}·(Pt·Cl)$_2$ or CB[7]^{Pt}·(Pt·Cl) (1.0 mM, 0.60 μmol, 0.60 mL) were treated with an aqueous solution of peptide FGGGC (50 mM, 0.60 μmol, 12 μL) and the mixture was subsequently stirred and kept at 40 ºC for 1 h. The color of the sample turned from yellow to red.

Assemblies CB[8]^{Pt}·(**1**·CB[7]^{Phe})$_2$, CB[8]^{Pt}·**1**$_2$·CB[8]$^{Phe}{}_{HT}$ and CB[7]^{Pt}·**1**·CB[7]^{Phe} were obtained by adding CB[7] or CB[8] in relevant amounts, and the mixtures were sonicated for 10 min.

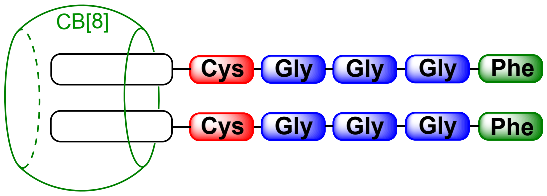

CB[8]^{Pt}·1$_2$. ^{1}H NMR (500 MHz, D$_2$O) δ 9.15 (dd, *J* = 10.6, 5.8 Hz, 4H, H^6), 8.58 (t, *J* = 8.0 Hz, 4H, H^3), 8.24 – 8.10 (m, 8H, H^4+H$^{3'}$), 7.58 (dt, *J* = 27.7, 6.6 Hz, 4H, H^5), 7.42 – 7.27 (m, 6H, H^i+H^j), 7.24 (d, *J* = 7.1 Hz, 4H, H^h), 6.67 – 6.47 (m, 6H, H^7+H^8), 5.77 (d, *J* = 15.3 Hz, 8H, H$^{CB[8]}$), 5.64 (d, *J* = 15.3 Hz, 8H, H$^{CB[8]}$), 5.38 (s, 16H, H$^{CB[8]}$), 4.40 (dd, *J* = 7.8, 4.3 Hz, 2H, H^b), 4.31 – 4.20 (m, 2H, H^f), 4.07 (t, *J* = 16.5 Hz, 16H, H$^{CB[8]}$), 3.97 – 3.83 (m, 8H, H^c+H^e), 3.76 (q, *J* = 16.8 Hz, 4H, H^d), 3.17 (ddt, *J* = 28.7, 13.2, 7.0 Hz, 4H, H^g), 3.01 – 2.67 (m, 4H, H^a). ^{13}C NMR (126 MHz, D$_2$O) δ 32.24, 36.77, 42.09, 42.38, 42.49, 53.45, 54.43, 56.58, 71.93, 106.03, 106.15, 109.02, 109.18, 109.24, 121.52, 121.60, 126.24, 126.28, 128.01, 129.02, 129.16, 129.38, 133.72, 137.24, 137.32, 137.40, 142.35, 151.28, 152.44, 152.56, 152.84, 155.79, 155.85, 156.30, 156.66, 156.84, 157.90, 161.90, 162.01, 163.90, 164.00, 169.83, 170.78, 171.38, 171.67, 174.34, 176.65. ^{19}F NMR (471 MHz, D$_2$O) δ -107.03. HRMS (ESI): m/z = 1644.42682 [M]$^{2+}$ (calcd. 1644.4224 for $C_{126}H_{124}F_4N_{48}O_{28}Pt_2S_2$). UV-Vis (H$_2$O) λ 248 (*ε* = 18.4 × 10^3), 279 (*ε* = 21.4 × 10^3), 317 (*ε* = 12.2 × 10^3), 374 (*ε* = 3.55 × 10^3), 574 (*ε* = 7.10 × 10^2).

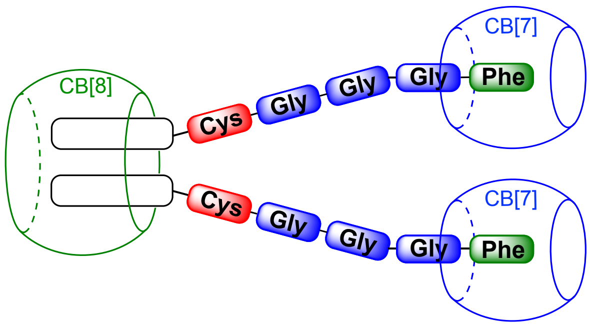

CB[8]^{Pt}·(1·CB[7]^{Phe})$_2$. ^{1}H NMR (500 MHz, D_2O) δ 9.08 (dd, *J* = 20.8, 5.6 Hz, 4H, H^6), 8.54 (t, *J* = 7.8 Hz, 4H, H^3), 8.29 – 8.00 (m, 8H, H^4+$H^{3'}$), 7.67 – 7.47 (m, 4H, H^5), 6.60 (s, 2H, H^j), 6.56 – 6.43 (m, 10H, H^7+H^8+H^i), 6.31 (s, 4H, H^h), 5.70 (d, *J* = 15.3 Hz, 8H, $H^{CB[8]}$), 5.65 – 5.52 (m, 36H, $H^{CB[8]}$+$H^{CB[7]}$), 5.35 (s, 28H, $H^{CB[7]}$), 5.34 (s, 16H, $H^{CB[8]}$), 4.39 (t, *J* = 5.9 Hz, 2H, H^b), 4.26 (d, *J* = 10.8 Hz, 2H, H^f), 4.19 – 3.96 (m, 48H, $H^{CB[8]}$+$H^{CB[7]}$+H^c), 3.94 – 3.76 (m, 4H, H^e), 3.75 – 3.55 (m, 4H, H^d), 3.05 (d, *J* = 14.4 Hz, 2H, H^g), 2.97 (d, *J* = 10.9 Hz, 2H, H^a), 2.84 – 2.73 (m, 2H, H^a), 2.49 (t, *J* = 13.6 Hz, 2H, H^g). ^{13}C NMR (126 MHz, D_2O) δ 174.22, 172.47, 171.64, 170.57, 170.35, 163.94, 162.03, 157.91, 156.88, 156.70, 156.25, 152.82, 152.44, 151.31, 142.42, 137.32, 133.66, 129.10, 128.00, 127.30, 126.35, 121.65, 109.23, 109.04, 106.20, 105.97, 71.95, 71.14, 56.46, 54.90, 53.48, 52.50, 43.48, 42.49, 41.93, 36.08, 32.09. ^{19}F NMR (471 MHz, D_2O) δ -106.91. HRMS (ESI): m/z = 1403.38324 $[M+2H]^{4+}$ (calcd. 1403.38286 for $C_{210}H_{208}F_4N_{104}O_{56}Pt_2S_2$). UV-Vis ($H_2O$) λ 248 ($\varepsilon$ = 20.1 × 10^3), 279 (ε = 23.6 × 10^3), 318 (ε = 12.1 × 10^3), 375 (ε = 3.78 × 10^3), 541 (ε = 8.30 × 10^2).

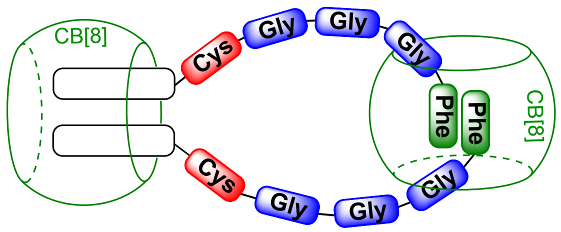

CB[8]^{Pt}·1$_2$·CB[8]$^{Phe}_{HT}$. ^{1}H NMR (500 MHz, D_2O) δ 9.29 (d, *J* = 5.6 Hz, 2H, H^6), 9.19 (d, *J* = 5.5 Hz, 2H, H^6), 8.70 (d, *J* = 8.0 Hz, 2H, H^3), 8.66 (d, *J* = 8.0 Hz, 2H, H^3), 8.41 – 8.10 (m, 8H, H^4+$H^{3'}$), 7.70 (q, *J* = 8.2, 7.7 Hz, 4H, H^5), 6.83 – 6.61 (m, 8H, H^7+H^8+H^j), 6.56 – 6.36 (m, 4H, H^i), 6.03 (d, *J* = 7.6 Hz, 4H, H^h), 5.86 (d, *J* = 15.3 Hz, 24H, $H^{CB[8]}$), 5.74 (d, *J* = 15.3 Hz, 8H, $H^{CB[8]}$), 5.64 (s, 16H, $H^{CB[8]}$), 5.48 (s, 16H, $H^{CB[8]}$), 4.53 (t, *J* = 6.2 Hz, H^b), 4.43 (d, *J* = 17.3 Hz, H^f), 4.30 (d, *J* = 15.2 Hz, 16H, $H^{CB[8]}$), 4.23 – 4.11 (m, 24H $H^{CB[8]}$+H^c+H^e), 4.01 – 3.85 (m, , 4H, H^d), 3.10 – 3.00 (m, 2H, H^a), 2.97 – 2.87 (m, 2H, H^a), 2.39 (d, *J* = 13.0 Hz, 2H, H^g), 2.25 (t, *J* = 13.2 Hz, 2H, H^g). ^{13}C NMR (176 MHz, D_2O) δ 174.22, 172.30, 171.77, 170.59, 170.24, 163.74, 163.67, 162.31, 162.24, 157.90, 157.87, 156.91, 156.89, 156.81, 156.78, 156.76, 156.69, 153.90, 152.95, 152.90, 152.86, 152.46, 151.26, 142.76, 142.40, 137.32, 132.78, 129.14, 129.06, 128.95, 127.77, 126.64, 126.33, 126.25, 121.60, 121.49, 109.21, 109.07, 106.33, 106.18, 106.03, 72.04, 71.95, 56.38, 54.97, 53.62, 53.47, 43.26, 42.63, 41.97, 37.16, 32.01. ^{19}F NMR (471 MHz, D_2O) δ -107.06. HRMS (ESI): m/z = 1538.73924 $[M+H]^{3+}$ (calcd. 1538.74316 for $C_{174}H_{171}F_4N_{80}O_{44}Pt_2S_2$). UV-Vis ($H_2O$) λ 249 ($\varepsilon$ = 18.4 × 10^3), 279 (ε = 22.5 × 10^3), 317 (ε = 12.3 × 10^3), 375 (ε = 3.66 × 10^3), 544 (ε = 9.13 × 10^2).

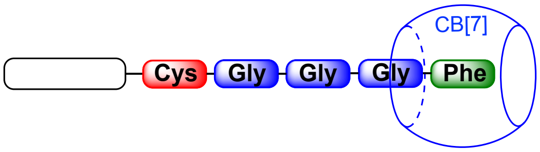

1·CB[7]^{Phe}. ^{1}H NMR (500 MHz, D_2O) δ 8.82 (s, 2H, H^6), 8.27 (s, 2H, H^3), 8.16 (s, 4H, $H^{4}+H^{3'}$), 7.57 (s, 2H, H^5), 7.25 (s, 2H, H^7), 7.01 (s, 1H, H^8), 6.72 (s, 1H, H^j), 6.64 (s, 2H, H^i), 6.50 (s, 2H, H^h), 5.58 (d, J = 15.4 Hz, 14H, $H^{CB[7]}$), 5.34 (s, 14H, $H^{CB[7]}$), 4.34 (s, 1H, H^b), 4.20 (d, J = 10.8 Hz, 1H, H^f), 4.04 (d, J = 15.4 Hz, 14H, $H^{CB[7]}$), 3.98 – 3.88 (m, 2H, H^c), 3.77 (q, J = 17.5, 17.0 Hz, 2H, H^e), 3.66 – 3.47 (m, 2H, H^d), 2.90 (dd, J = 47.7, 14.5 Hz, 2H, H^g), 2.60 (dt, J = 56.2, 13.0 Hz, 2H, H^a). ^{13}C NMR (176 MHz, D_2O) δ 174.45, 172.10, 171.42, 170.33, 170.31, 164.07, 164.00, 162.66, 162.58, 157.80, 156.23, 153.36, 152.42, 151.19, 142.51, 137.66, 133.64, 129.33, 128.28, 127.82, 127.66, 125.72, 110.57, 71.13, 56.84, 54.72, 52.48, 43.13, 42.48, 41.87, 36.28, 31.81. ^{19}F NMR (471 MHz, D_2O) δ -107.86. HRMS (ESI): m/z = 1071.28488 $[M+H]^{2+}$ (calcd. 1071.28463 for $C_{81}H_{80}F_2N_{36}O_{20}Pt_1S_1$). HRMS (MALDI): m/z = 2141.569 $[M]^+$ (calcd. 2141.562 for $C_{81}H_{79}F_2N_{36}O_{20}Pt_1S_1$). UV-Vis ($H_2O$) λ 250 ($\varepsilon$ = 23.5 × 10^3), 289 (ε = 27.4 × 10^3), 348 (ε = 8.54 × 10^3), 500 (ε = 1.06 × 10^3).

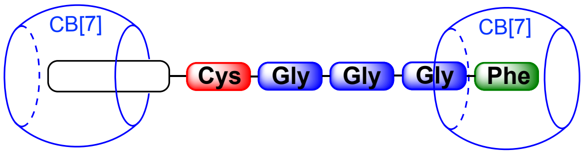

CB[7]^{Pt}·1·CB[7]^{Phe}. ^{1}H NMR (500 MHz, D_2O) δ 9.19 (d, J = 4.6 Hz, 2H, H^6), 9.15 (d, J = 8.0 Hz, 2H, H^3), 8.67 (s, 2H, $H^{3'}$), 8.40 (t, J = 7.7 Hz, 2H, H^4), 7.79 (t, J = 6.8 Hz, 2H, H^5), 6.76 (d, J = 7.1 Hz, 2H, H^7), 6.65 (t, J = 7.5 Hz, 1H, H^8), 6.58 – 6.45 (m, 3H, $H^{i}+H^{j}$), 6.34 (d, J = 7.5 Hz, 2H, H^h), 5.73 (d, J = 15.4 Hz, 7H, $H^{CB[7]}$), 5.67 (d, J = 15.5 Hz, 7H, $H^{CB[7]}$), 5.62 (dd, J = 15.2, 4.3 Hz, 14H, $H^{CB[7]}$), 5.40 (d, J = 4.8 Hz, 28H, $H^{CB[7]}$), 4.57 – 4.53 (m, 1H, H^b), 4.39 – 4.29 (m, 1H, H^f), 4.17 (s, 2H, H^c), 4.15 – 4.03 (m, 28H, $H^{CB[7]}$), 4.02 – 3.57 (m, 4H, $H^{d}+H^{e}$), 3.04 (d, J = 13.0 Hz, 2H, H^g), 2.98 – 2.89 (m, 1H, H^a), 2.58 – 2.43 (m, 1H, H^a). ^{13}C NMR (176 MHz, D_2O) δ 174.26, 172.56, 171.63, 170.56, 163.72, 163.64, 162.30, 162.22, 159.85, 156.36, 156.29, 156.21, 156.14, 153.46, 151.77, 142.18, 139.45, 133.65, 128.54, 127.98, 127.94, 127.53, 127.23, 127.19, 126.63, 123.70, 109.31, 109.28, 109.18, 109.15, 103.93, 71.18, 71.13, 71.11, 55.85, 54.89, 52.53, 52.50, 52.47, 46.51, 43.55, 42.47, 42.00, 36.06. ^{19}F NMR (471 MHz, D_2O) δ -109.96. HRMS (ESI): m/z = 1652.45424 $[M+H]^{2+}$ (calcd. 1652.45652 for $C_{123}H_{122}F_2N_{64}O_{34}Pt_1S_1$). UV-Vis ($H_2O$) λ 260 ($\varepsilon$ = 24.0 × 10^3), 289 (ε = 31.4 × 10^3), 345 (ε = 10.6 × 10^3), 484 (ε = 1.02 × 10^3).

4. ^{1}H and ^{13}C NMR spectra of the Pt/peptide/CB[n] assemblies

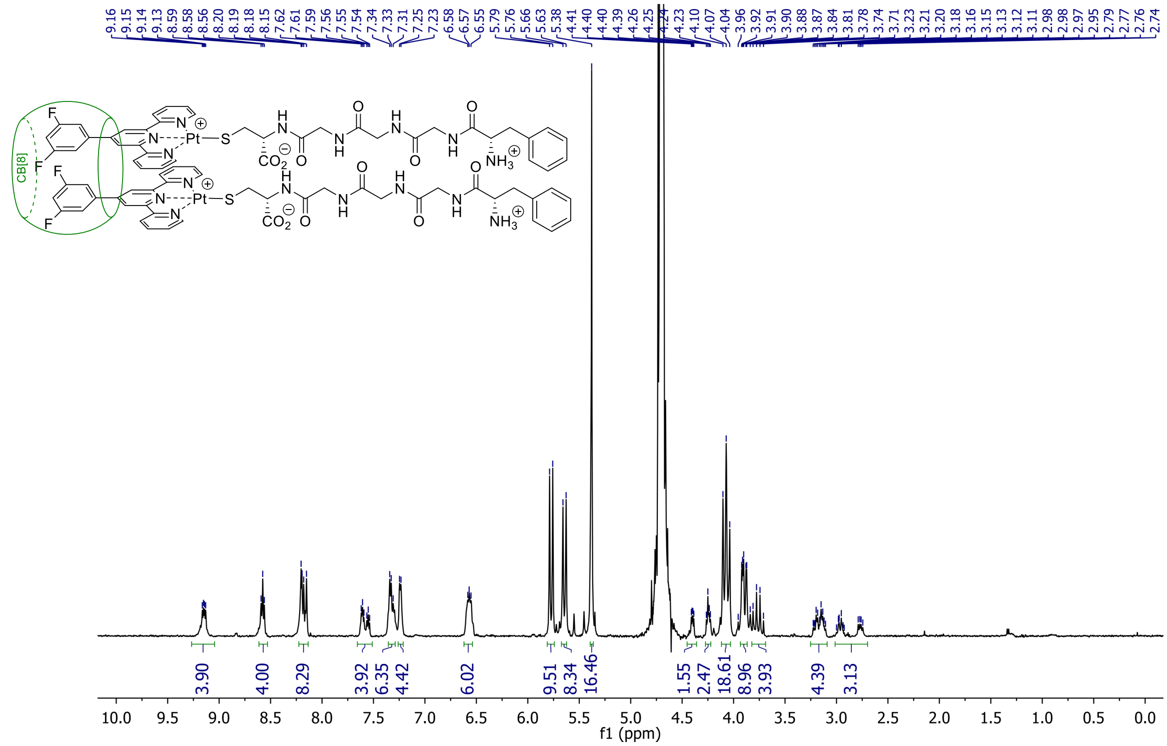

Figure S1. ^{1}H-NMR spectrum of assembly CB[8]^{Pt}·**1**$_2$ in D_2O.

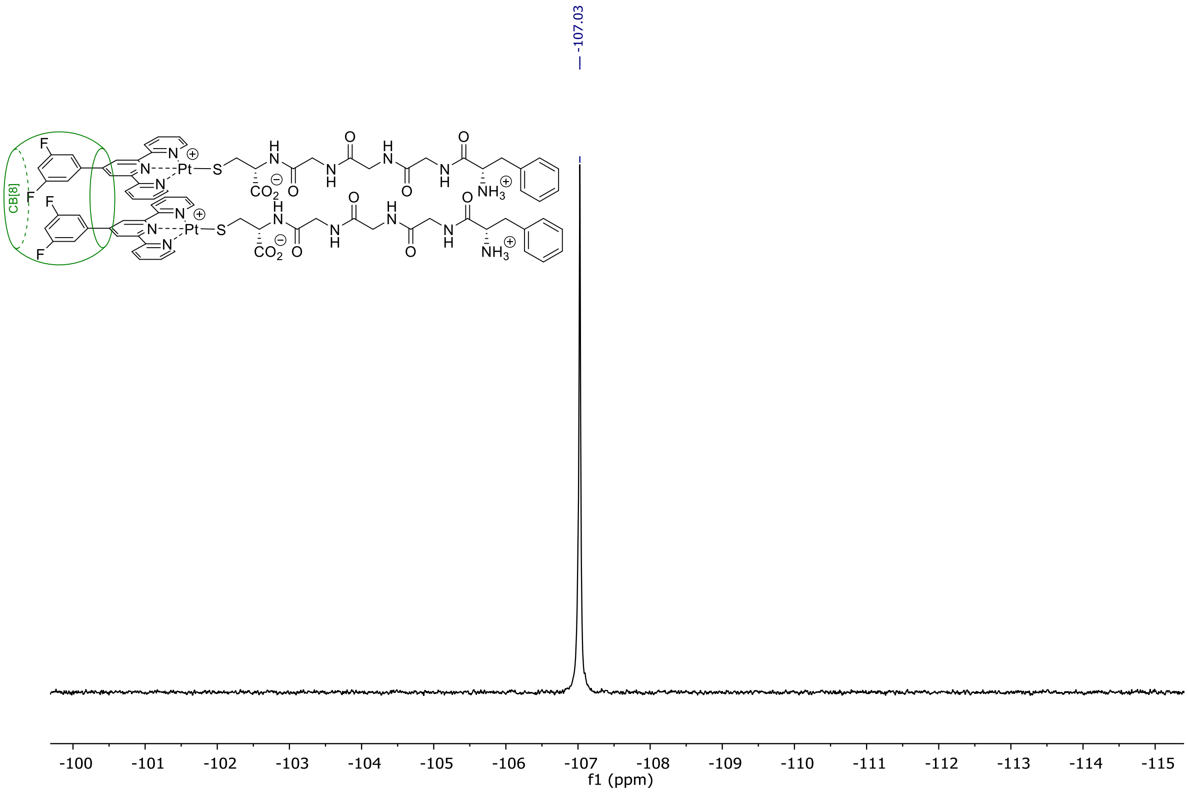

Figure S2. ^{19}F{^{1}H}-NMR spectrum of assembly CB[8]^{Pt}·**1**$_2$ in D_2O.

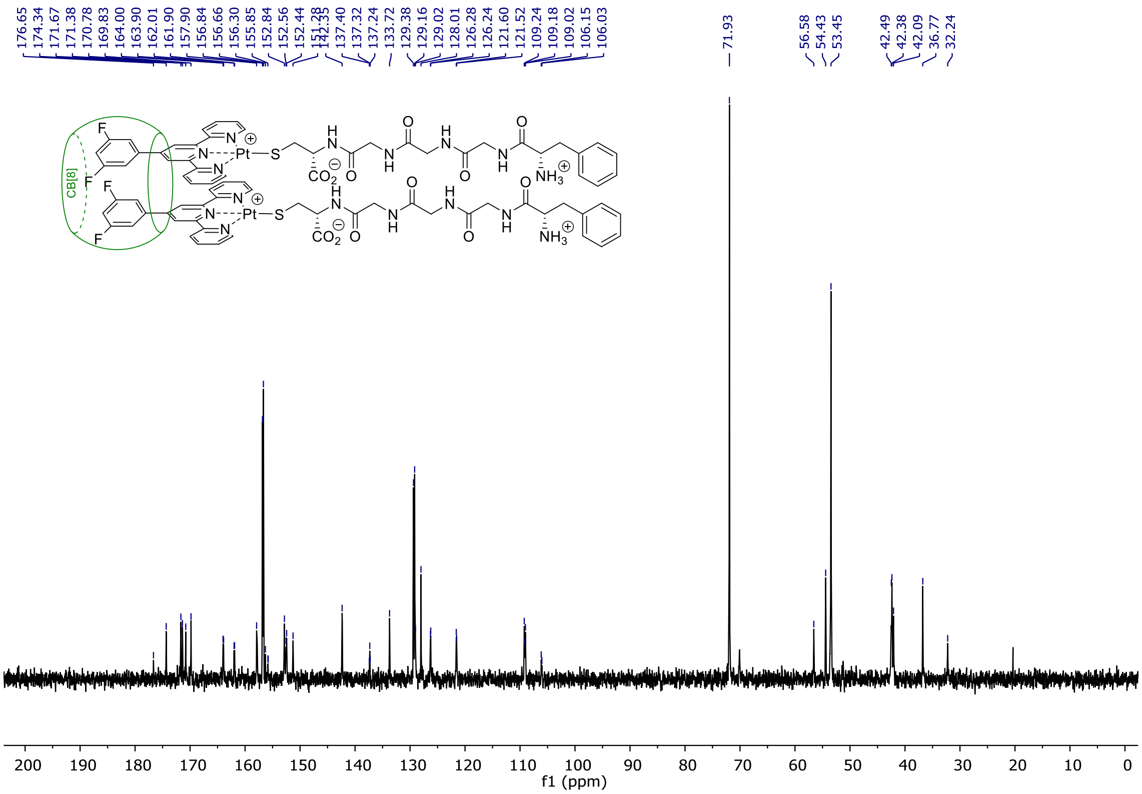

Figure S3. $^{13}C\{^{1}H\}$-NMR spectrum of assembly CB[8]$^{Pt}\cdot\mathbf{1}_2$ in D_2O.

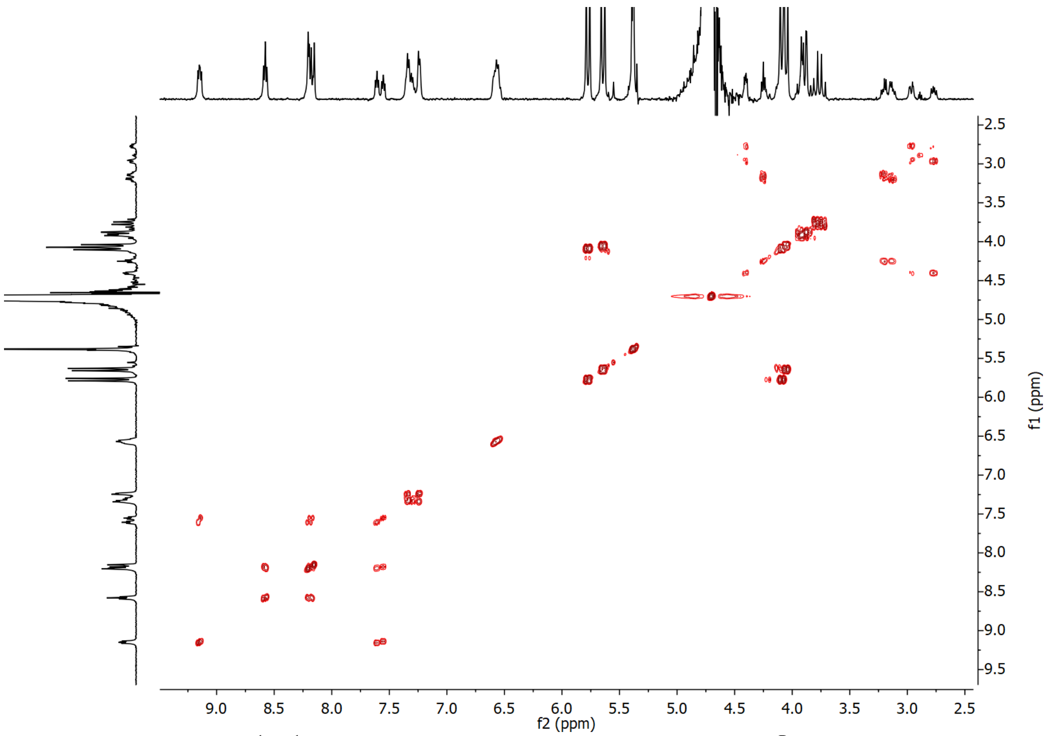

Figure S4. ^{1}H-^{1}H COSY NMR spectrum of assembly CB[8]$^{Pt}\cdot\mathbf{1}_2$ in D_2O.

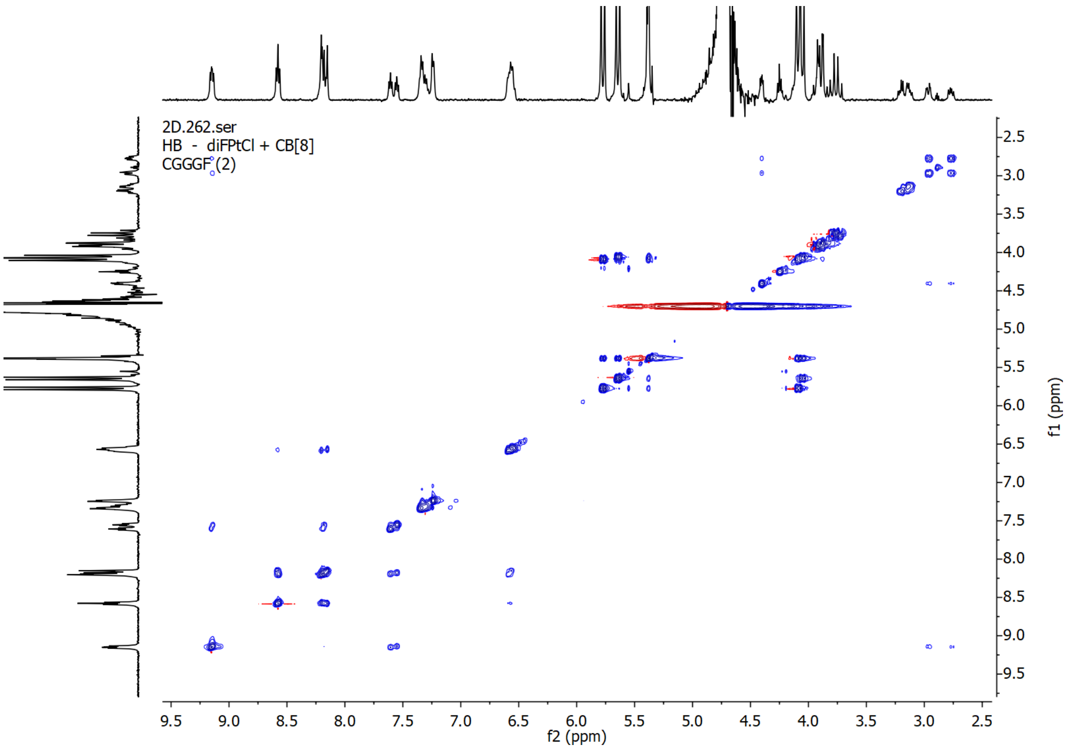

Figure S5. ^{1}H-^{1}H NOESY NMR spectrum of assembly CB[8]^{Pt}·**1**$_2$ in D_2O.

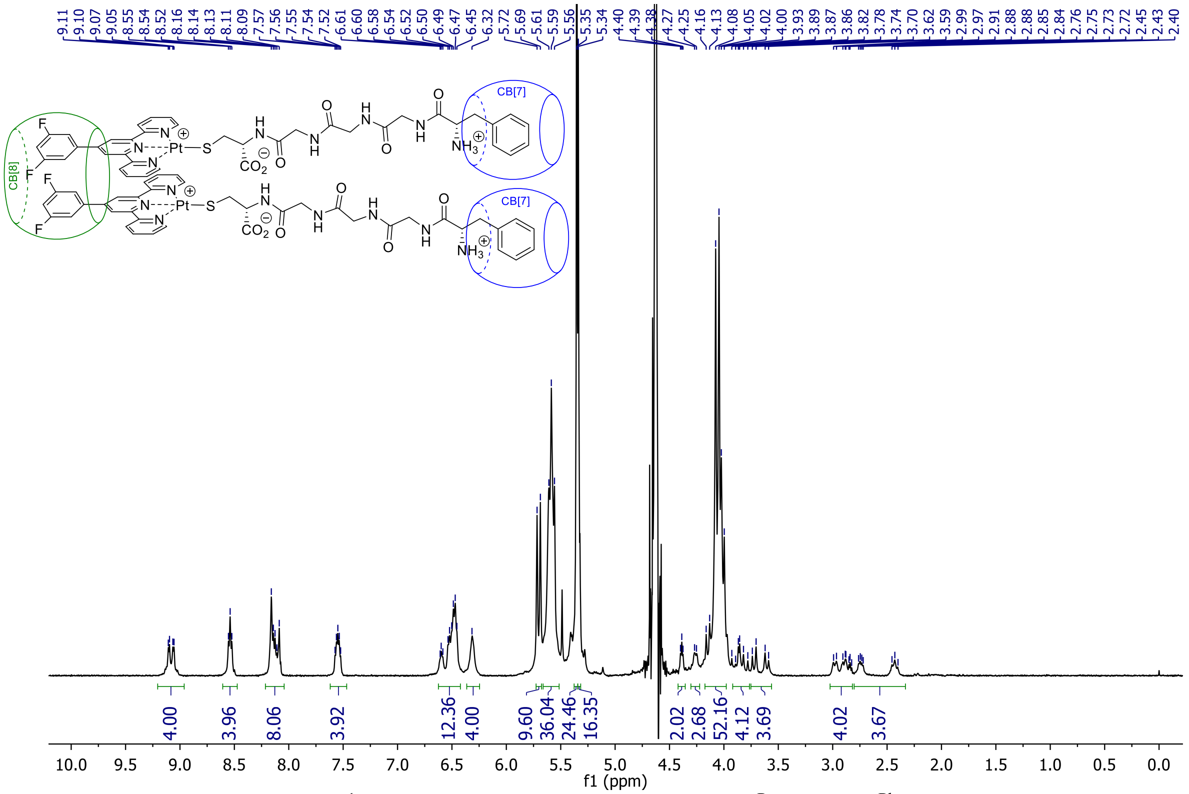

Figure S6. ^{1}H-NMR spectrum of assembly CB[8]^{Pt}·(**1**·CB[7]^{Phe})$_2$ in D_2O.

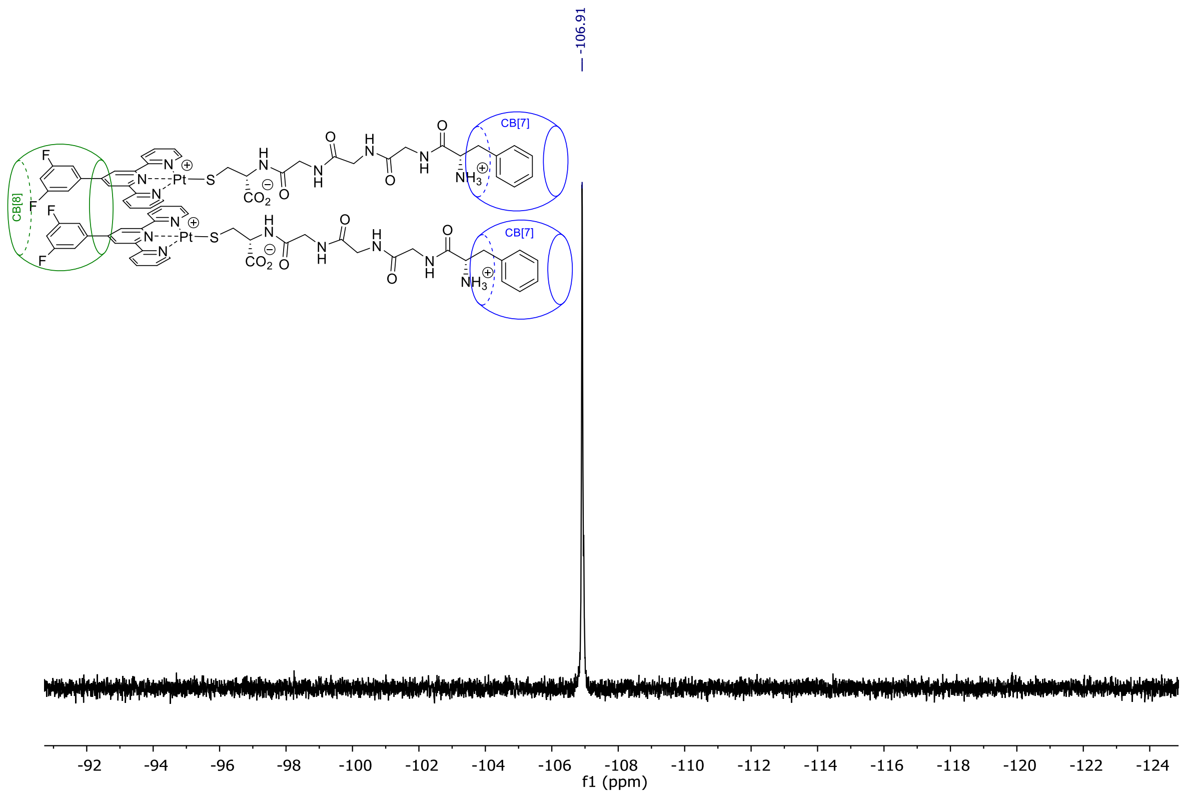

Figure S7. $^{19}F\{^{1}H\}$-NMR spectrum of assembly $CB[8]^{Pt}\cdot(\mathbf{1}\cdot CB[7]^{Phe})_2$ in D_2O.

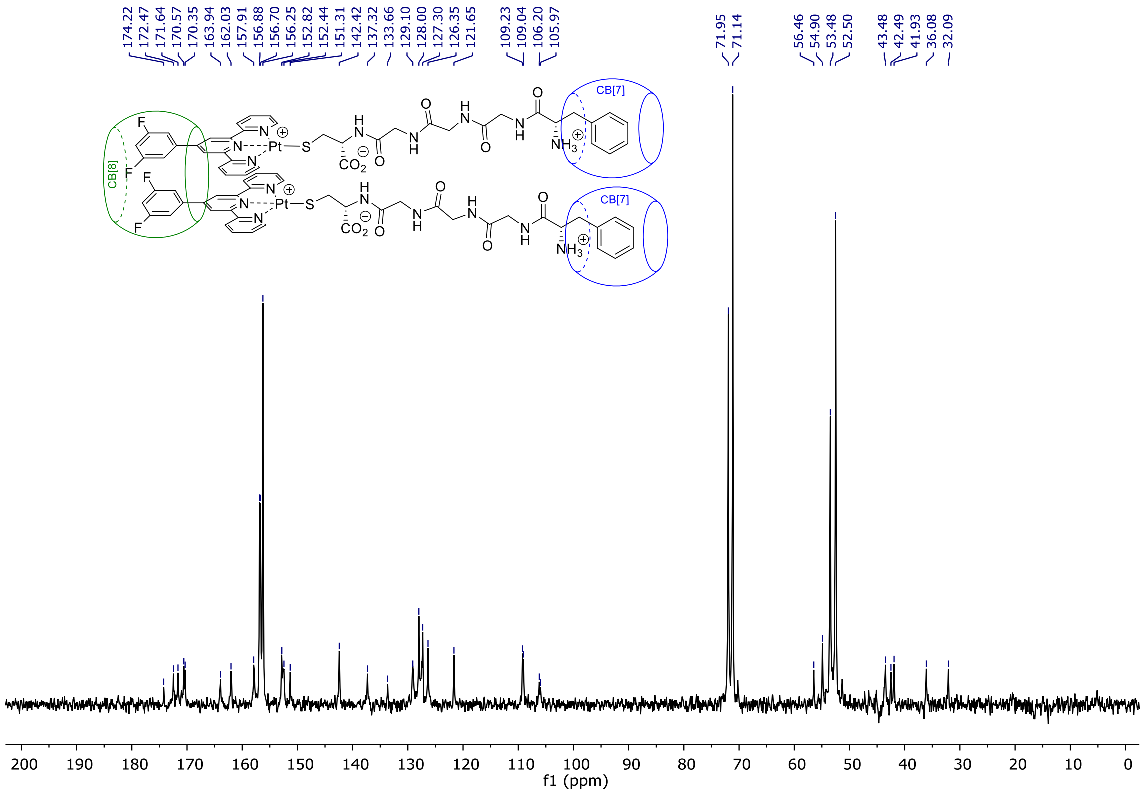

Figure S8. $^{13}C\{^{1}H\}$-NMR spectrum of assembly $CB[8]^{Pt}\cdot(\mathbf{1}\cdot CB[7]^{Phe})_2$ in D_2O.

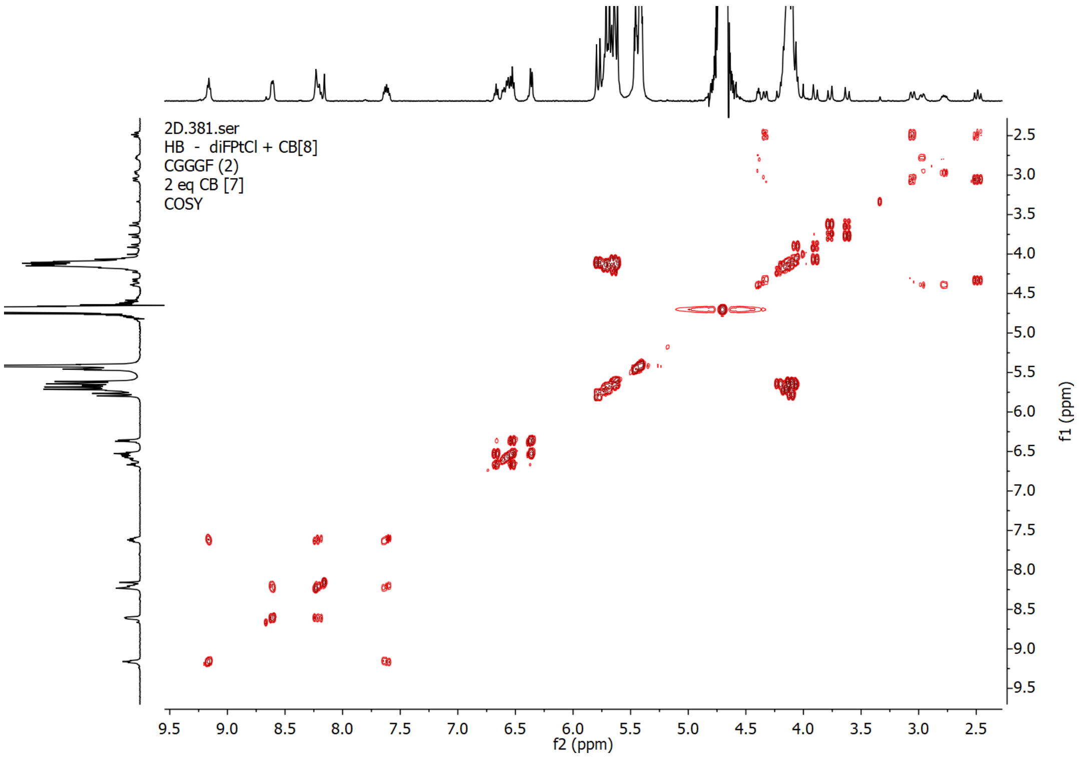

Figure S9. ^{1}H-^{1}H COSY NMR spectrum of assembly CB[8]^{Pt}·(**1**·CB[7]^{Phe})$_2$ in D_2O.

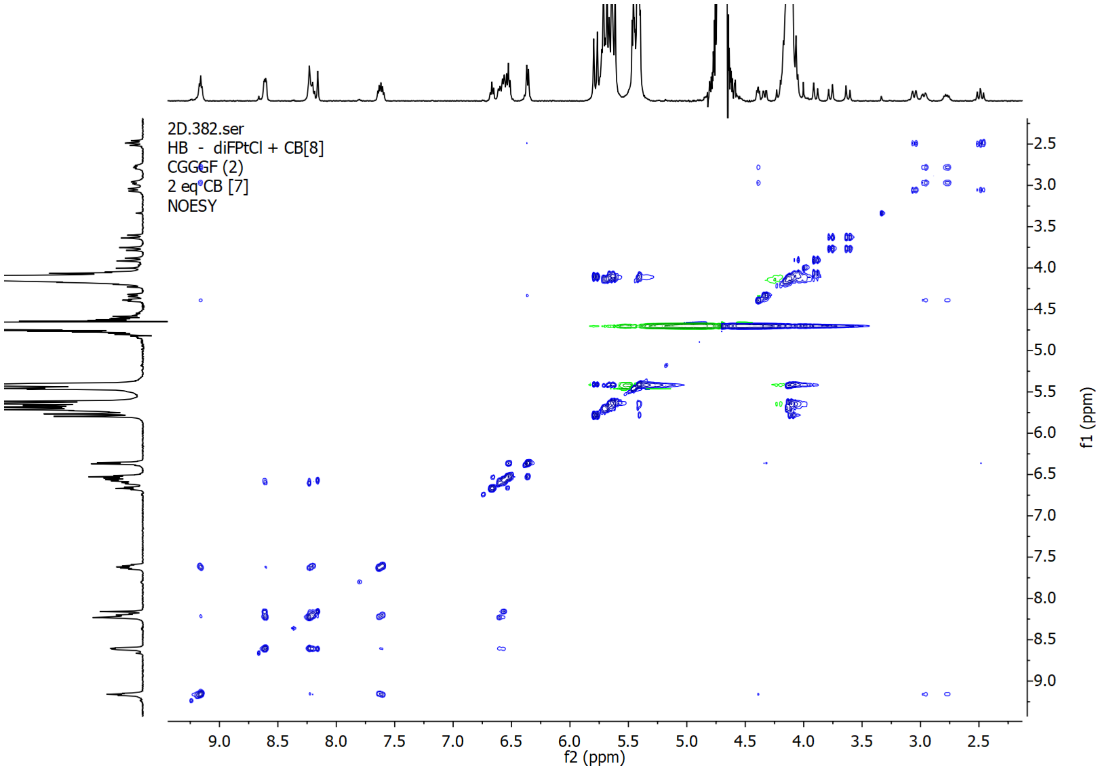

Figure S10. ^{1}H-^{1}H NOESY NMR spectrum of assembly CB[8]^{Pt}·(**1**·CB[7]^{Phe})$_2$ in D_2O.

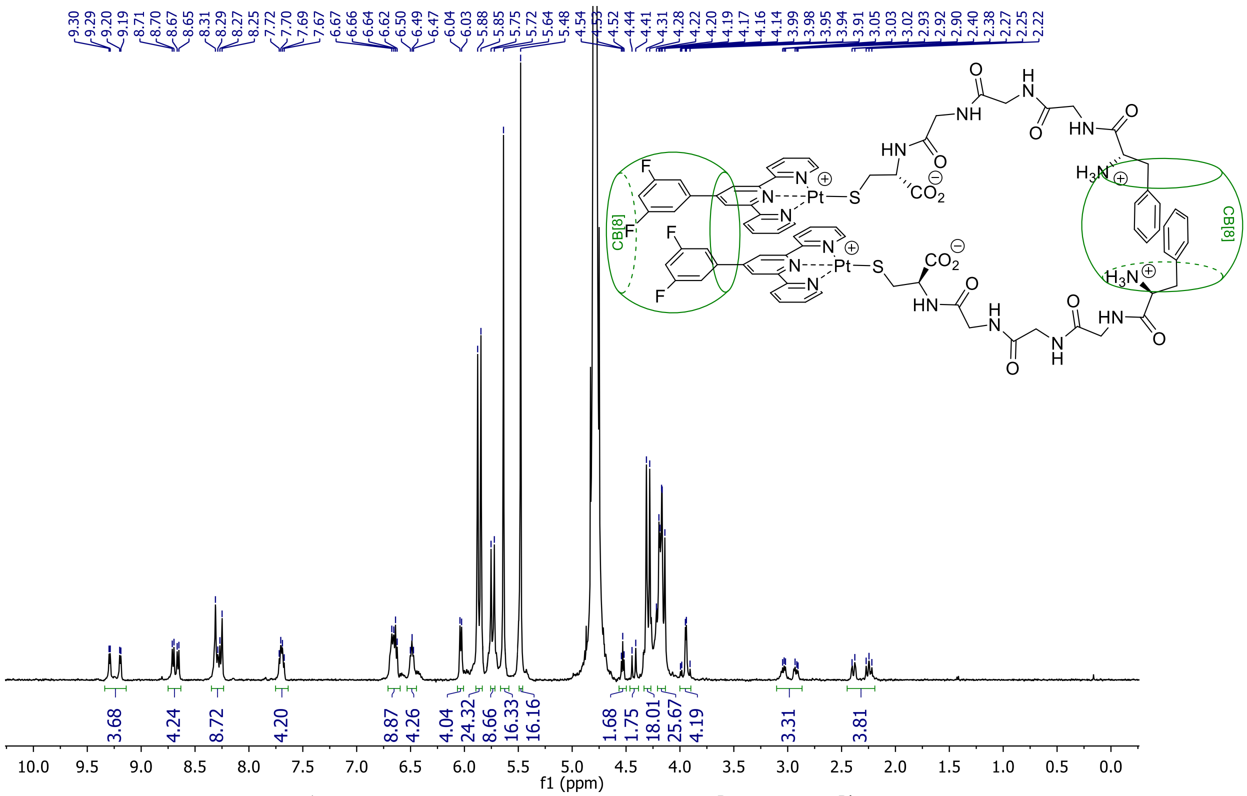

Figure S11. ^{1}H-NMR spectrum of assembly CB[8]^{Pt}·**1**$_2$·CB[8]$^{Phe}{}_{HT}$ in D_2O.

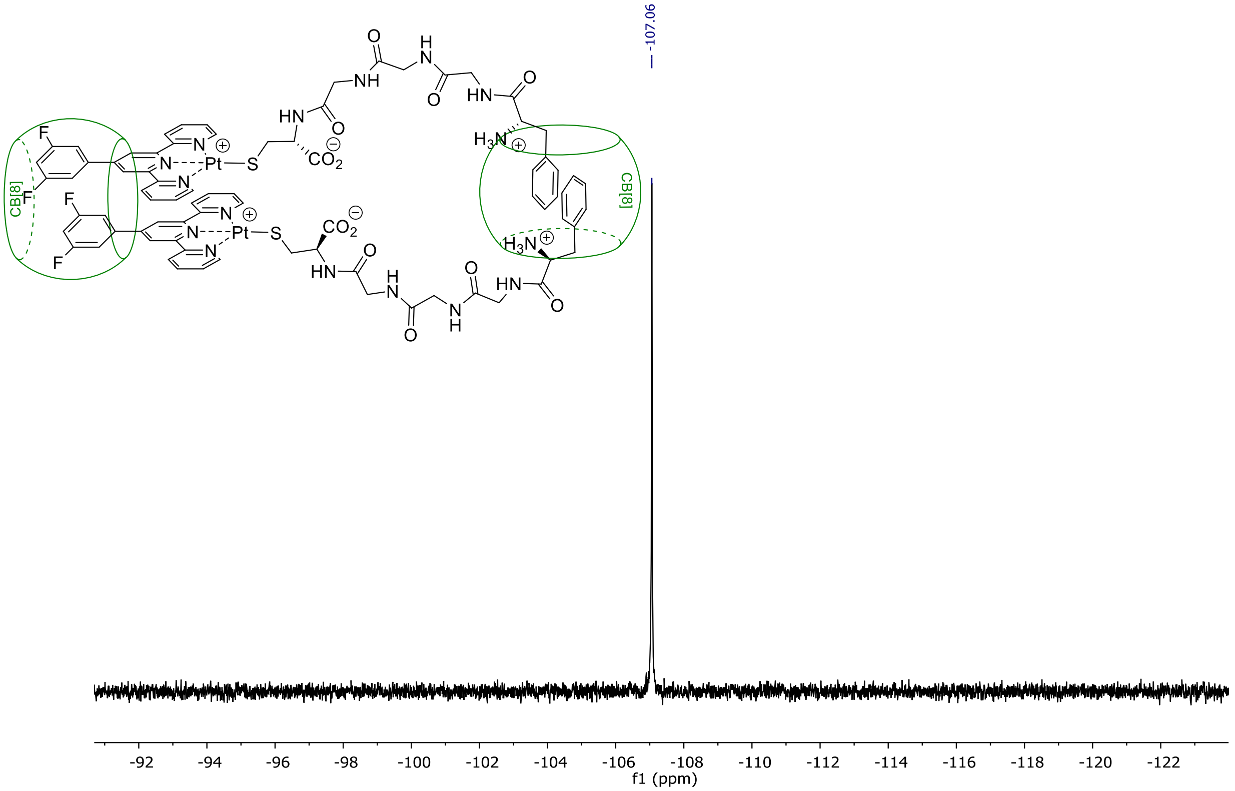

Figure S12. ^{19}F{^{1}H}-NMR spectrum of assembly CB[8]^{Pt}·**1**$_2$·CB[8]$^{Phe}{}_{HT}$ in D_2O.

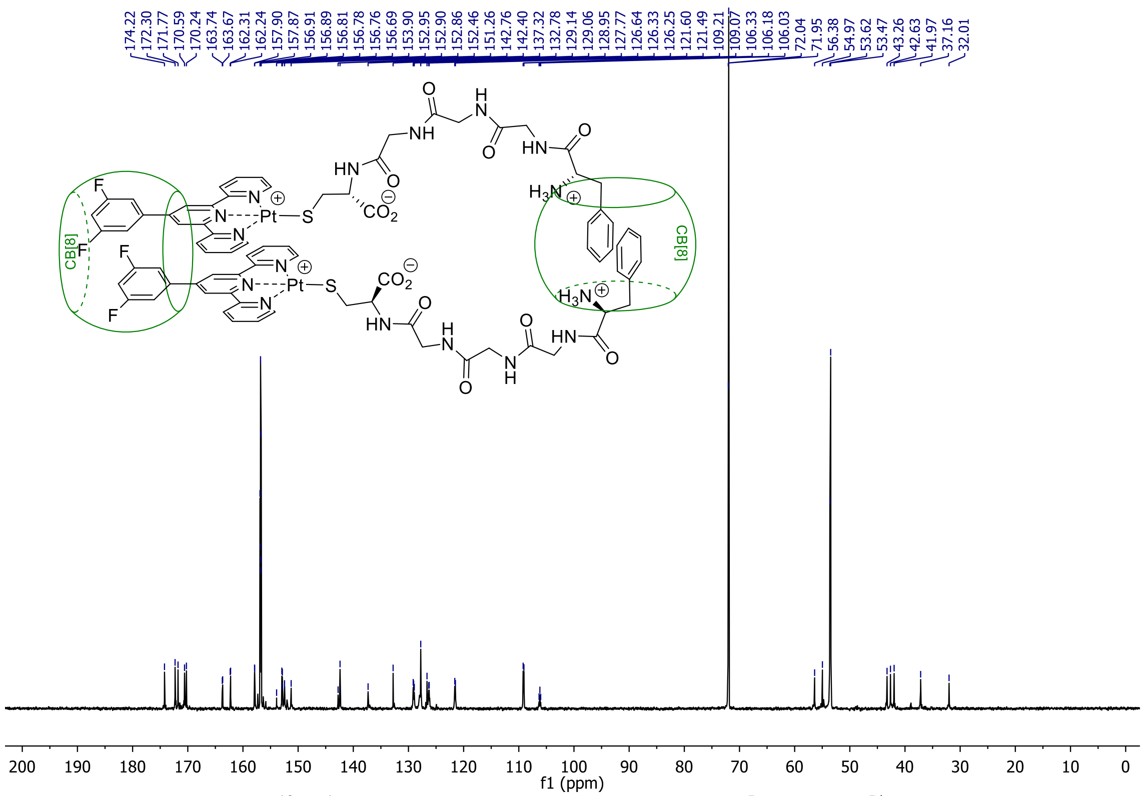

Figure S13. $^{13}C\{^{1}H\}$-NMR spectrum of assembly CB[8]^{Pt}·**1**$_2$·CB[8]$^{Phe}{}_{HT}$ in D_2O.

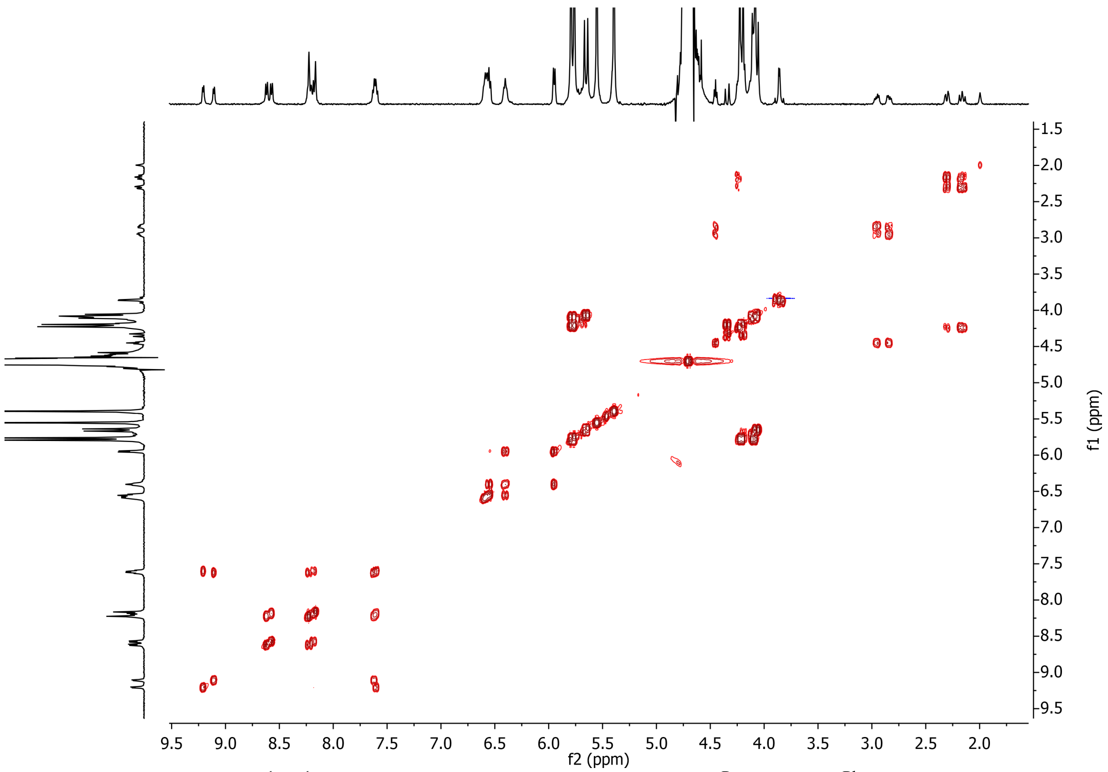

Figure S14. ^{1}H-^{1}H COSY spectrum of assembly CB[8]^{Pt}·**1**$_2$·CB[8]$^{Phe}{}_{HT}$ in D_2O.

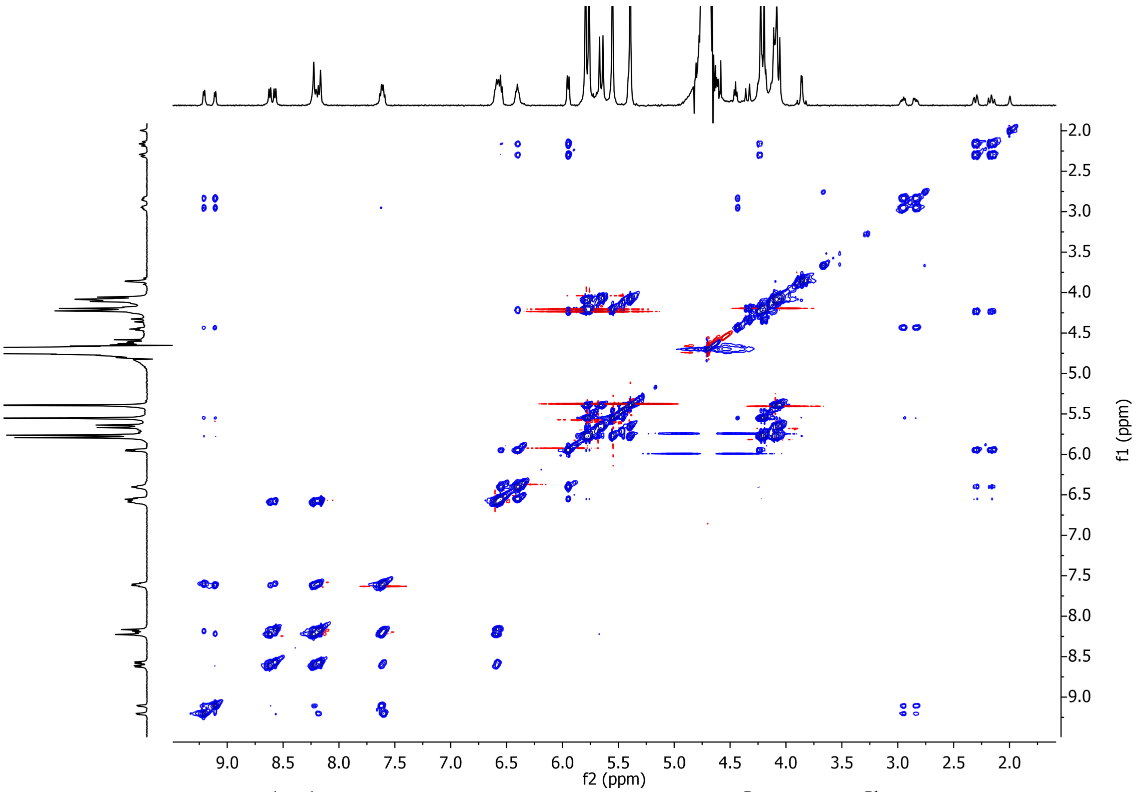

Figure S15. ^{1}H-^{1}H NOESY spectrum of assembly CB[8]^{Pt}·**1**$_2$·CB[8]$^{Phe}{}_{HT}$ in D_2O.

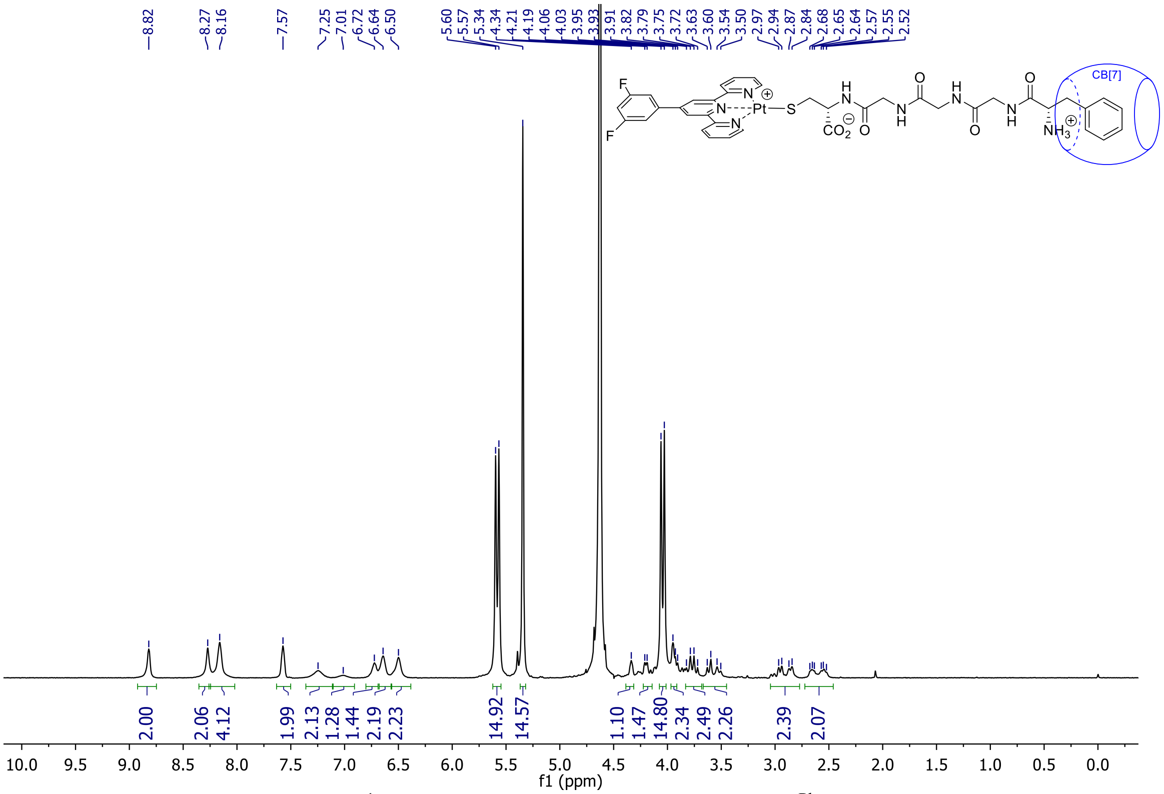

Figure S16. ^{1}H-NMR spectrum of assembly **1**·CB[7]^{Phe} in D_2O.

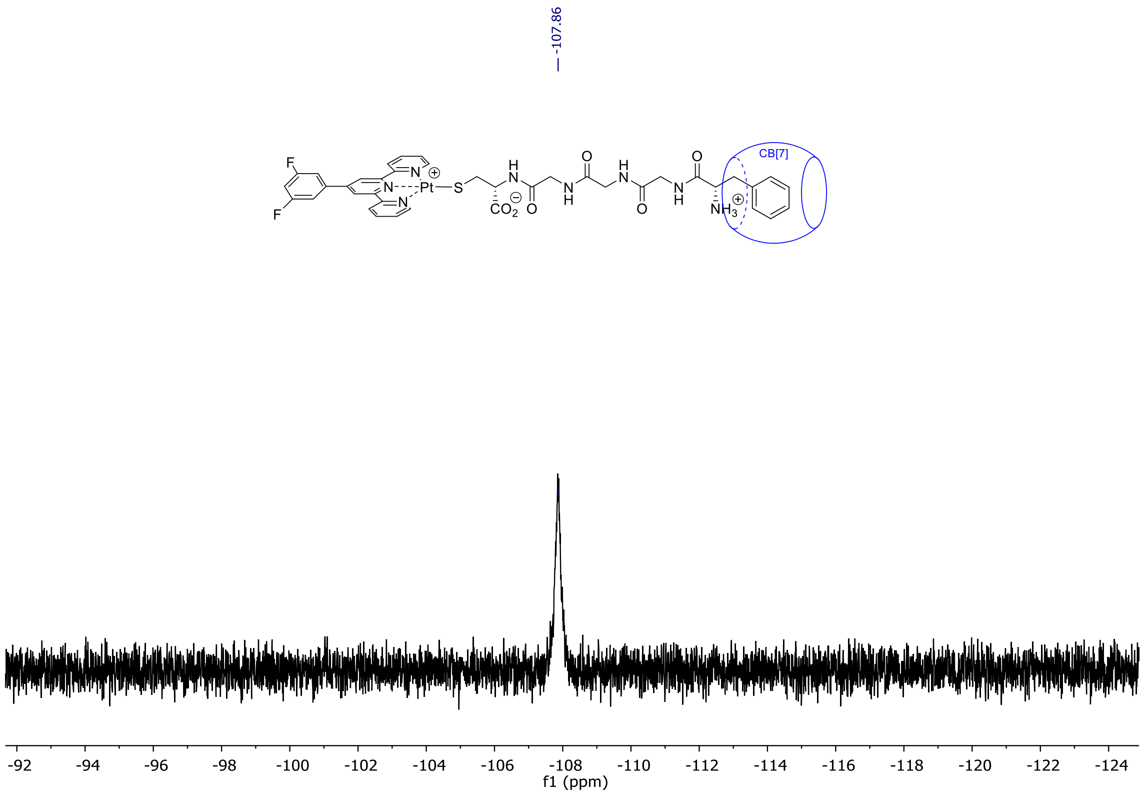

Figure S17. $^{19}F\{^{1}H\}$-NMR spectrum of **1**·CB[7][Phe] assembly in D_2O.

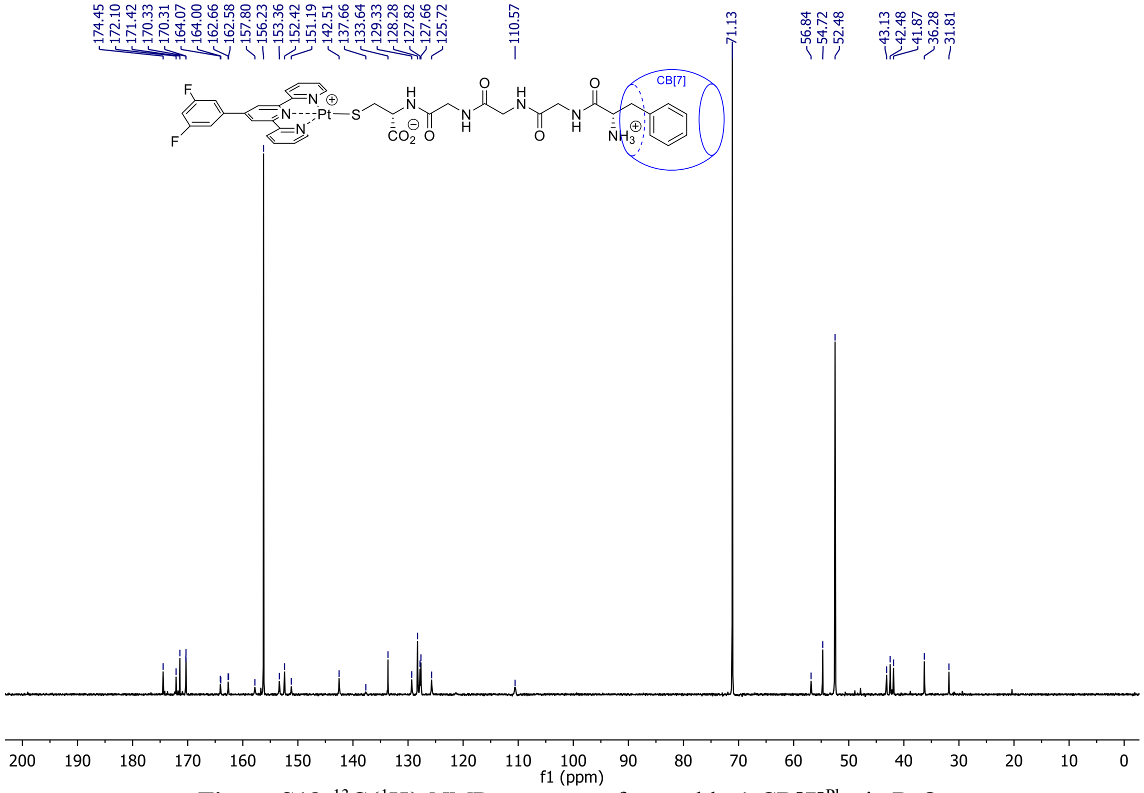

Figure S18. $^{13}C\{^{1}H\}$-NMR spectrum of assembly **1**·CB[7][Phe] in D_2O.

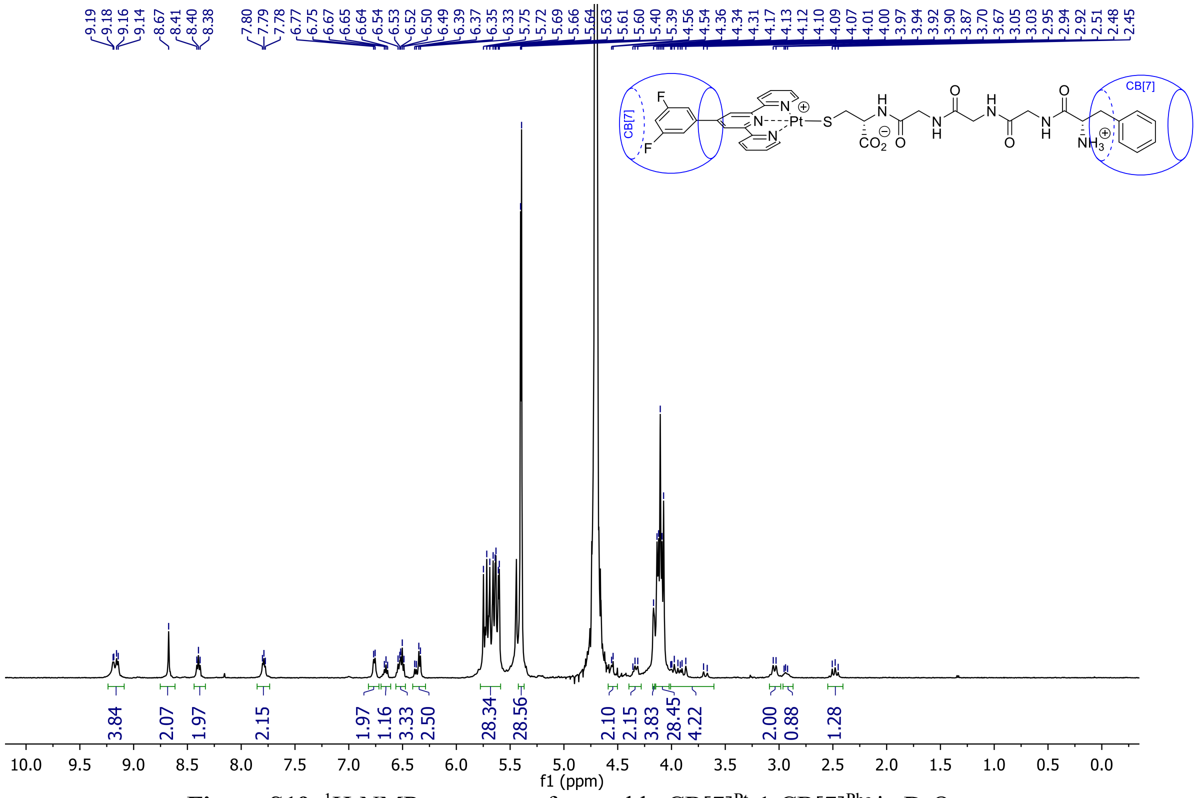

Figure S19. ^{1}H-NMR spectrum of assembly CB[7]^{Pt}·**1**·CB[7]^{Phe} in D_2O.

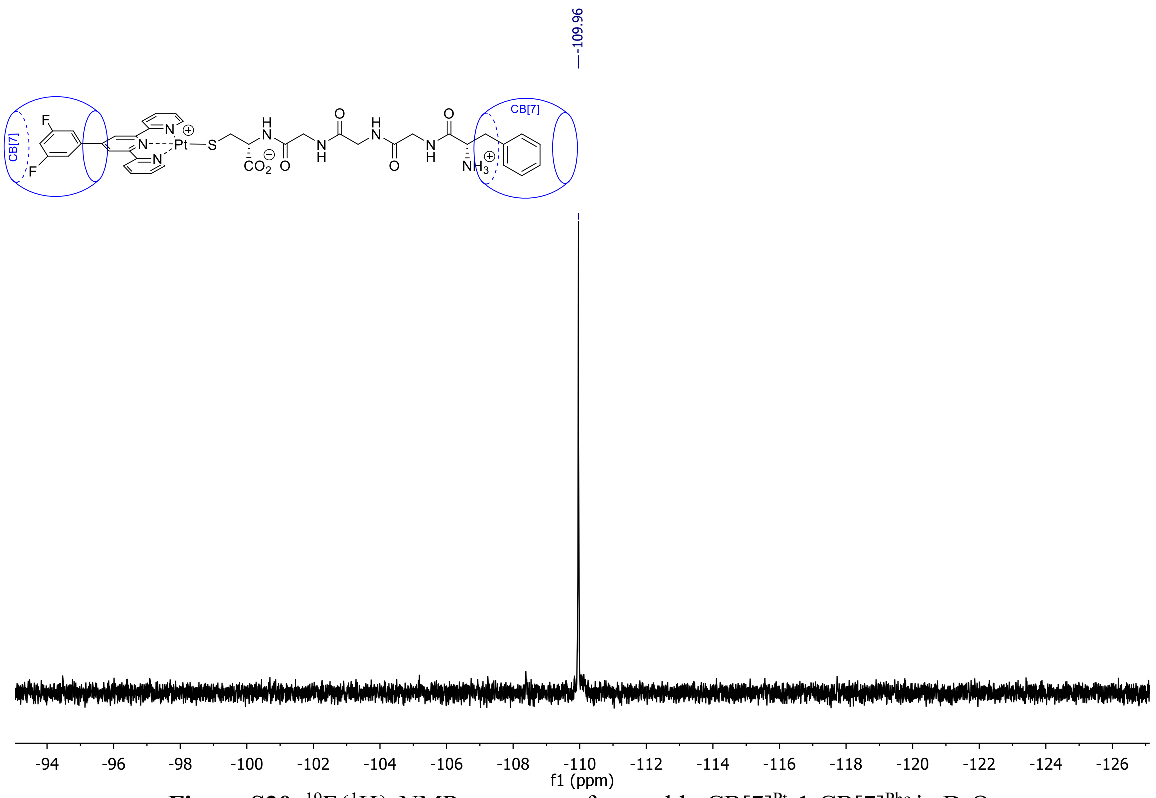

Figure S20. $^{19}F\{^{1}H\}$-NMR spectrum of assembly CB[7]^{Pt}·**1**·CB[7]^{Phe} in D_2O.

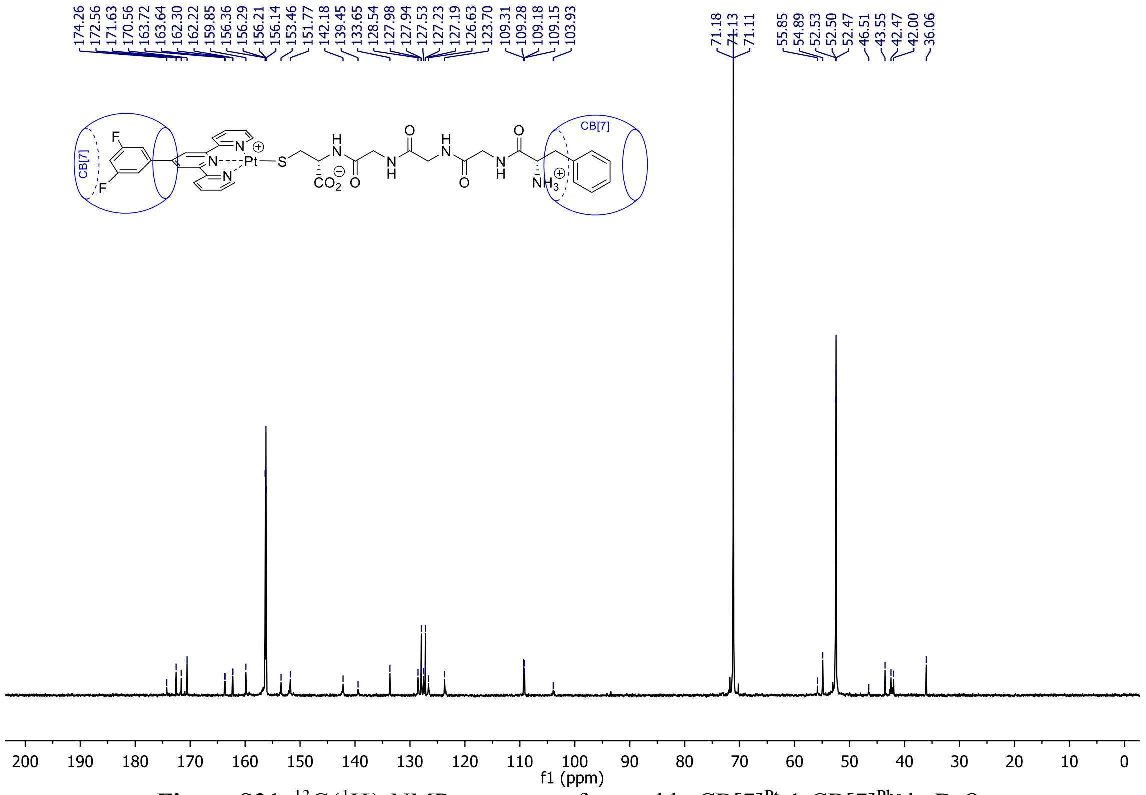

Figure S21. $^{13}C\{^1H\}$-NMR spectrum of assembly CB[7]^{Pt}·**1**·CB[7]^{Phe} in D_2O.

5. DOSY analysis for assemblies CB[8]^{Pt}·$\mathbf{1}_2$ and CB[8]^{Pt}·$\mathbf{1}_2$·CB[8]$^{Phe}_{HT}$

Diffusion constants obtained by DOSY experiments[4–6] were calculated by means of ^{1}H-NMR signals intensity attenuation upon field gradient application, fitted according to the Stejskal–Tanner Equation (1):[4,7]

$$I = I_0 e^{-D\gamma^2 g^2 \delta^2 (\Delta - \delta/3)} \quad (1)$$

Magnetic field gradient length (δ) was set to 1.5 ms and diffusion delay (Δ) to 0.1 s. The experimental setup consisted of 19 spectra with a gradient strength variation of 5% between two consecutive experiments.

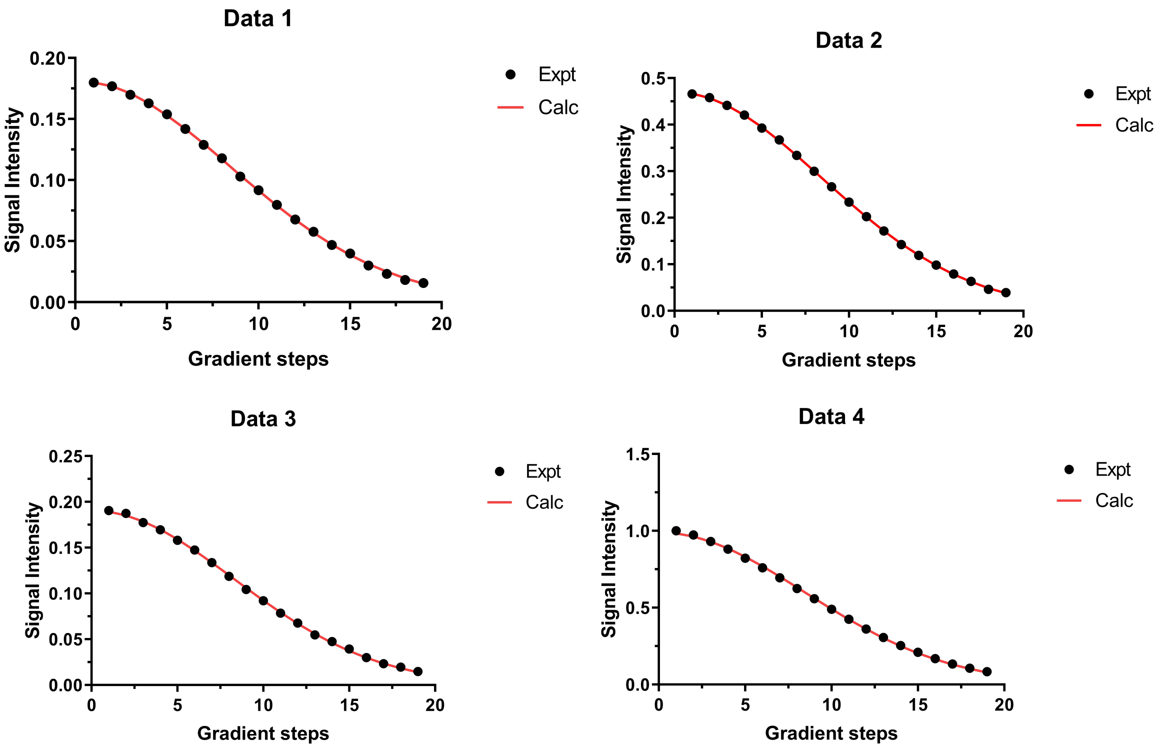

Figure S22. Selected fitted plots of signal attenuation for assembly CB[8]^{Pt}·**1**$_2$. Black dots: experimental values; red line: fitted curve.

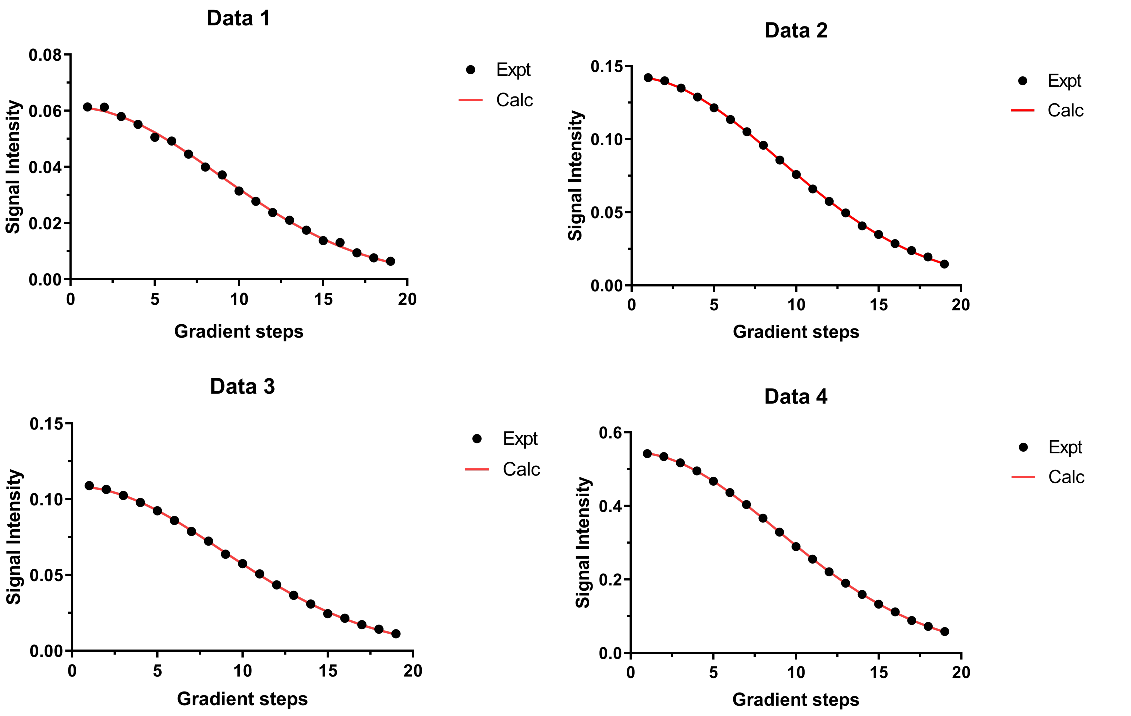

Figure S23. Selected fitted plots of signal attenuation for assembly CB[8]^{Pt}·**1**$_2$·CB[8]$^{Phe}{}_{HT}$. Black dots: experimental values; red line: fitted curve.

Entry	$CB[8]^{Pt}\cdot\mathbf{1}_2$	$CB[8]^{Pt}\cdot\mathbf{1}_2\cdot CB[8]^{Phe}_{HT}$
1	1.612×10^{-10}	1.489×10^{-10}
2	1.619×10^{-10}	1.507×10^{-10}
3	1.643×10^{-10}	1.524×10^{-10}
4	1.663×10^{-10}	1.520×10^{-10}
5	1.657×10^{-10}	1.479×10^{-10}
6	1.704×10^{-10}	1.516×10^{-10}
7	1.660×10^{-10}	1.509×10^{-10}
8	1.663×10^{-10}	1.515×10^{-10}
9	1.674×10^{-10}	1.492×10^{-10}
10	1.650×10^{-10}	1.474×10^{-10}
11	1.531×10^{-10}	1.478×10^{-10}
12	1.522×10^{-10}	1.464×10^{-10}
13	1.516×10^{-10}	1.481×10^{-10}
14	1.492×10^{-10}	1.544×10^{-10}
15	1.505×10^{-10}	1.472×10^{-10}
16	1.435×10^{-10}	1.500×10^{-10}
17	1.501×10^{-10}	1.492×10^{-10}
18	1.460×10^{-10}	1.508×10^{-10}
19		1.594×10^{-10}
20		1.568×10^{-10}
Average	$1.584 (\pm 0.035) \times 10^{-10}$	$1.506 (\pm 0.013) \times 10^{-10}$

Table S1. List of diffusion constants for individual proton signals in assemblies $CB[8]^{Pt}\cdot\mathbf{1}_2$ and $CB[8]^{Pt}\cdot\mathbf{1}_2\cdot CB[8]^{Phe}_{HT}$ obtained by fitting each signal attenuation upon gradient field application.

6. Mass spectrometry analysis of Pt/peptide/CB[n] assemblies

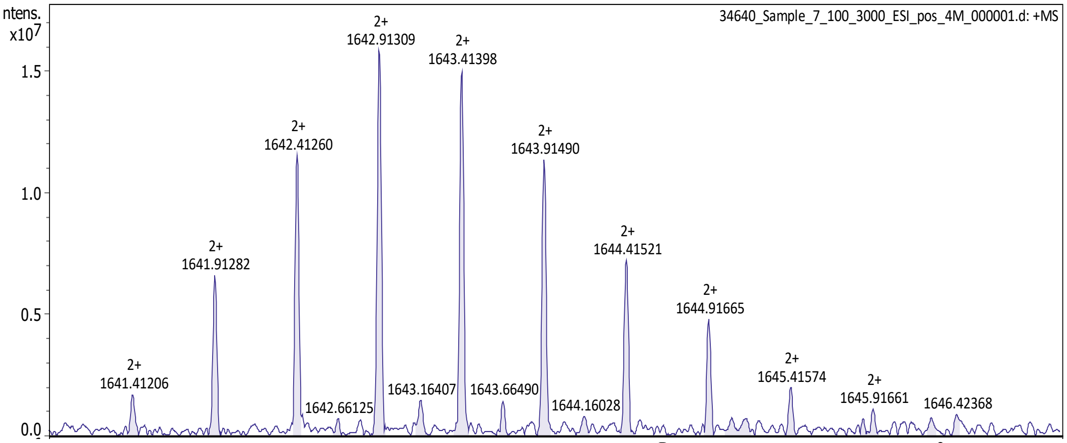

Figure S24. MS spectrum (ESI) of assembly CB[8]^{Pt}·$\mathbf{1}_2$ corresponding to $[M]^{2+}$.

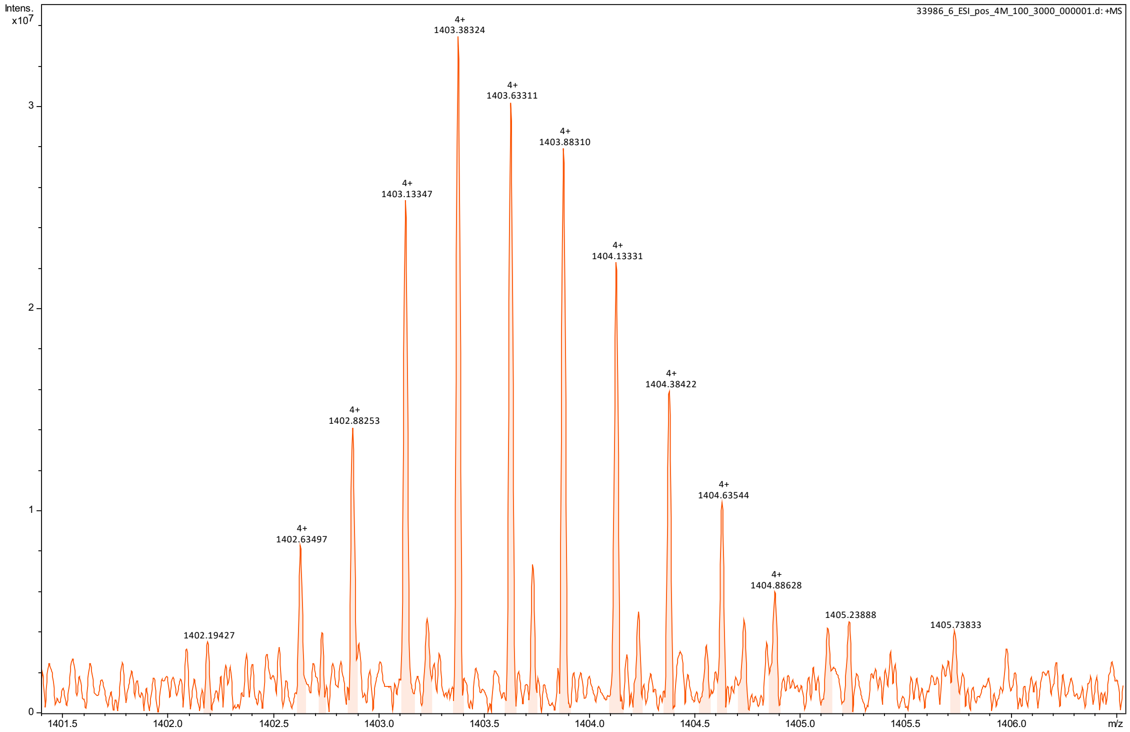

Figure S25. MS spectrum (ESI) of assembly CB[8]^{Pt}·($\mathbf{1}$·CB[7]^{Phe})$_2$ corresponding to $[M+2H]^{4+}$.

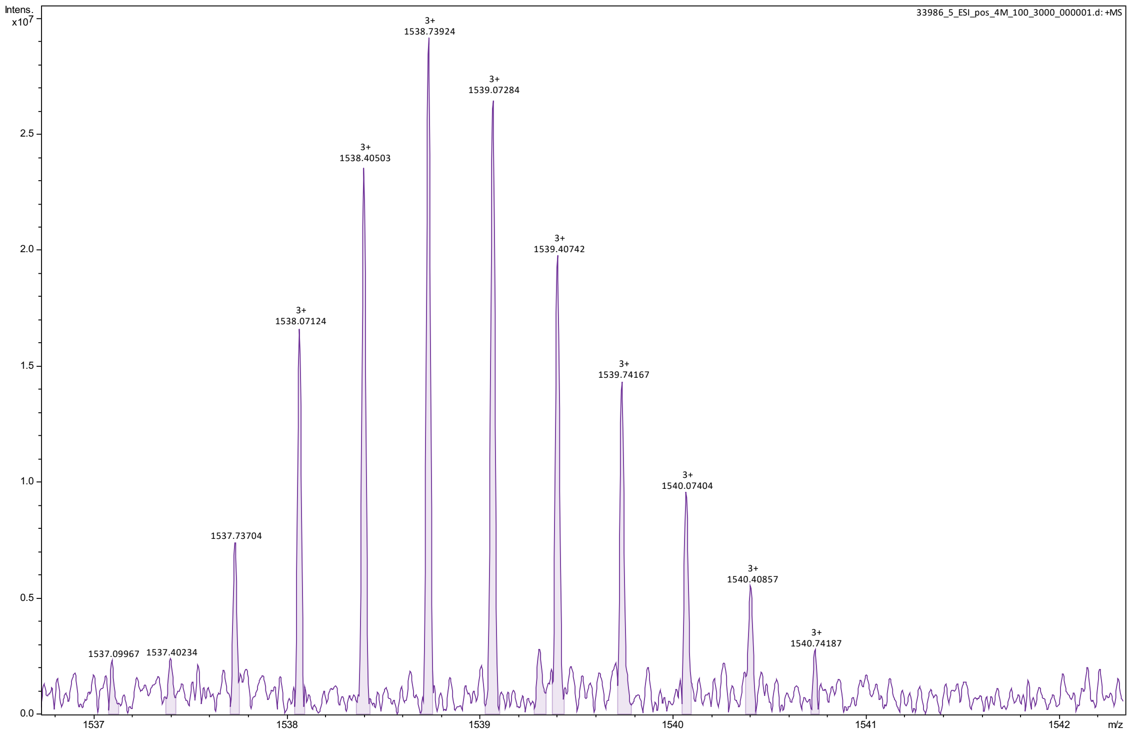

Figure S26. MS spectrum (ESI) of assembly CB[8]^{Pt}·**1**$_2$·CB[8]$^{Phe}{}_{HT}$ corresponding to [M+H]$^{3+}$.

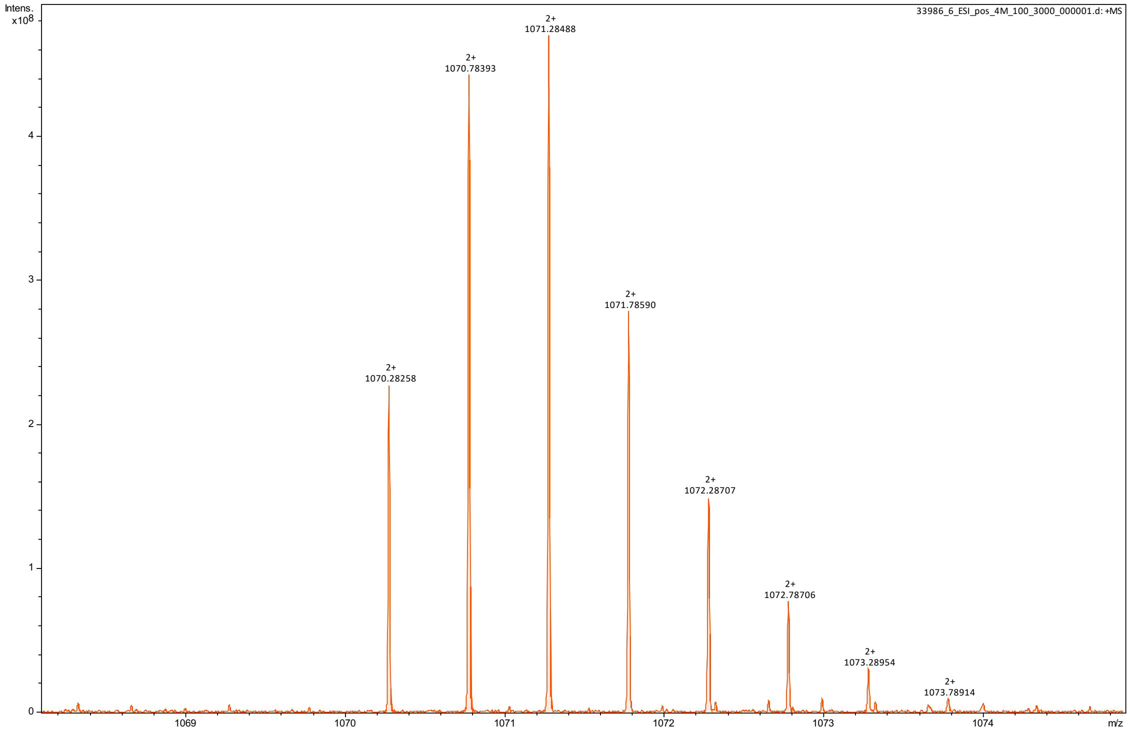

Figure S27. MS spectrum (ESI) of **1**·CB[7]^{Phe} assembly corresponding to [M+H]$^{2+}$.

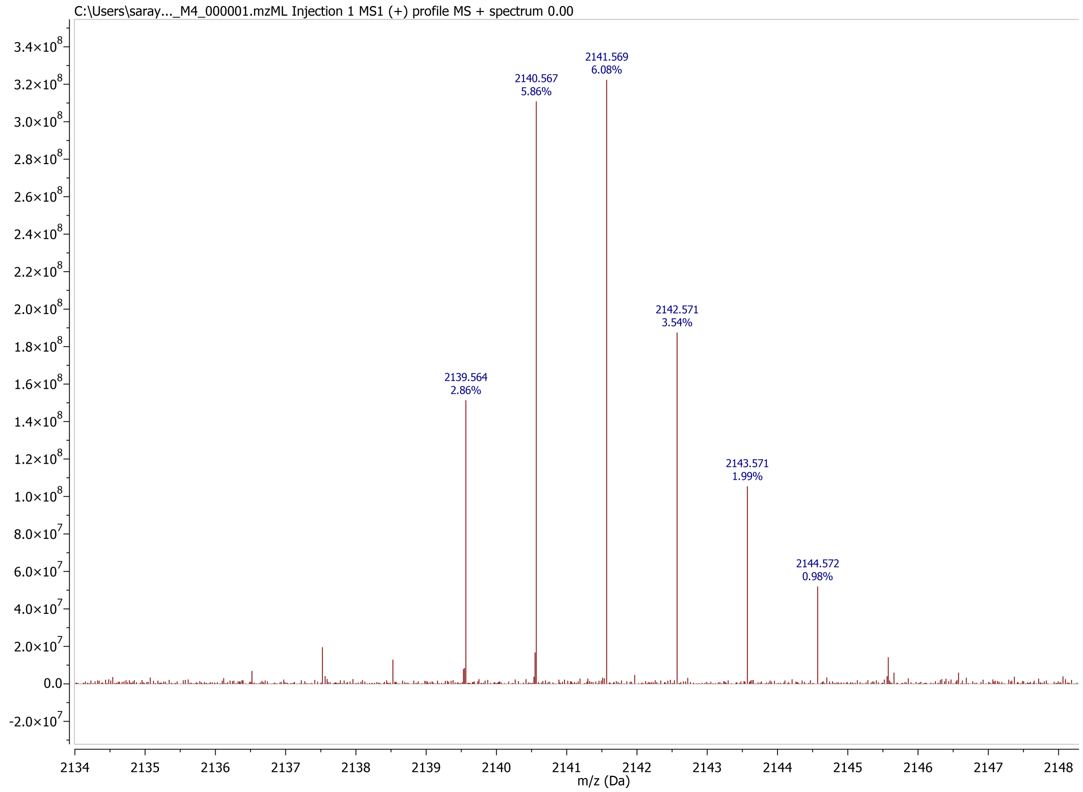

Figure S28. MS spectrum (MALDI) of **1**·CB[7]^{Phe} assembly corresponding to $[M]^+$.

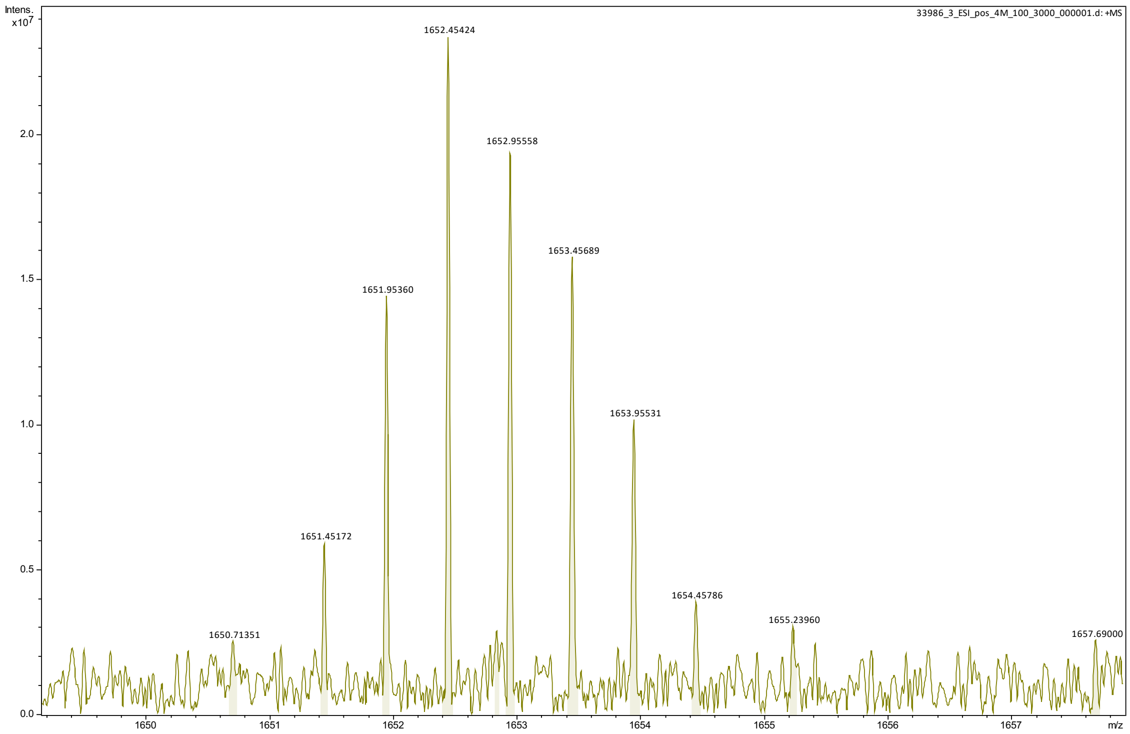

Figure S29. MS spectrum (ESI) of assembly CB[7]^{Pt}·**1**·CB[7]^{Phe} corresponding to $[M+H]^{2+}$.

7. UV-Vis absorption spectra of the Pt/peptide/CB[n] assemblies

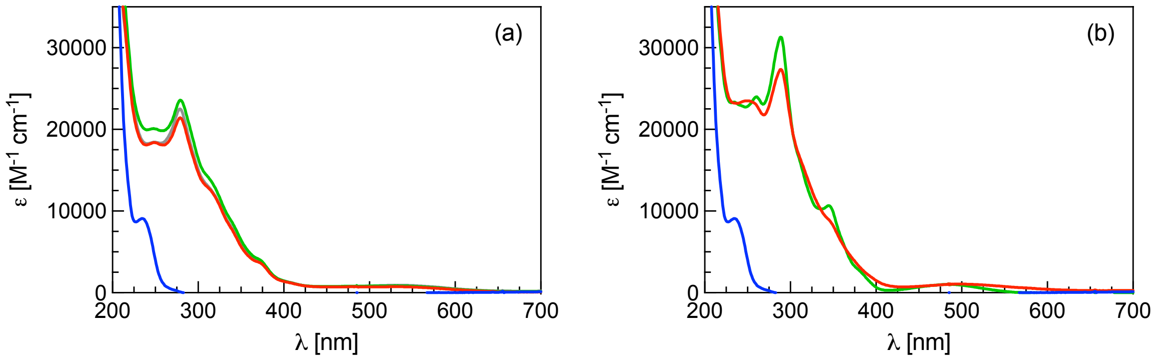

Figure S30. UV-Vis absorption spectra of (a) peptide FGGGC (in blue), and assemblies CB[8]^{Pt}·**1**$_2$ (in red), CB[8]^{Pt}·(**1**·CB[7]^{Phe})$_2$ (in green), and CB[8]^{Pt}·**1**$_2$·CB[8]^{Phe}$_{HT}$ (in grey); (b) peptide FGGGC (in blue), (b) **1**·CB[7]^{Phe} assemblies (in red), and (c) complex CB[7]^{Pt}·**1**·CB[7]^{Phe} (in green). All spectra recorded in H_2O; Pt concentration 50 μM.

8. Titration of peptide FGGGC with CB[8]

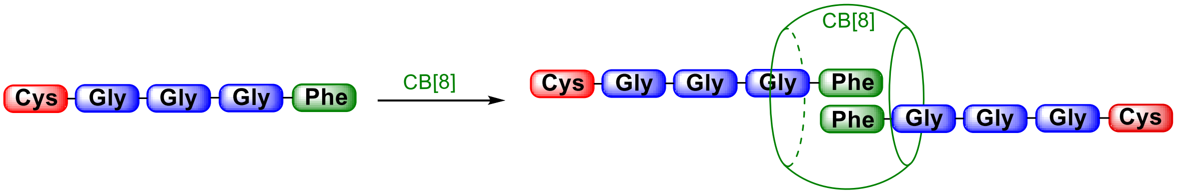

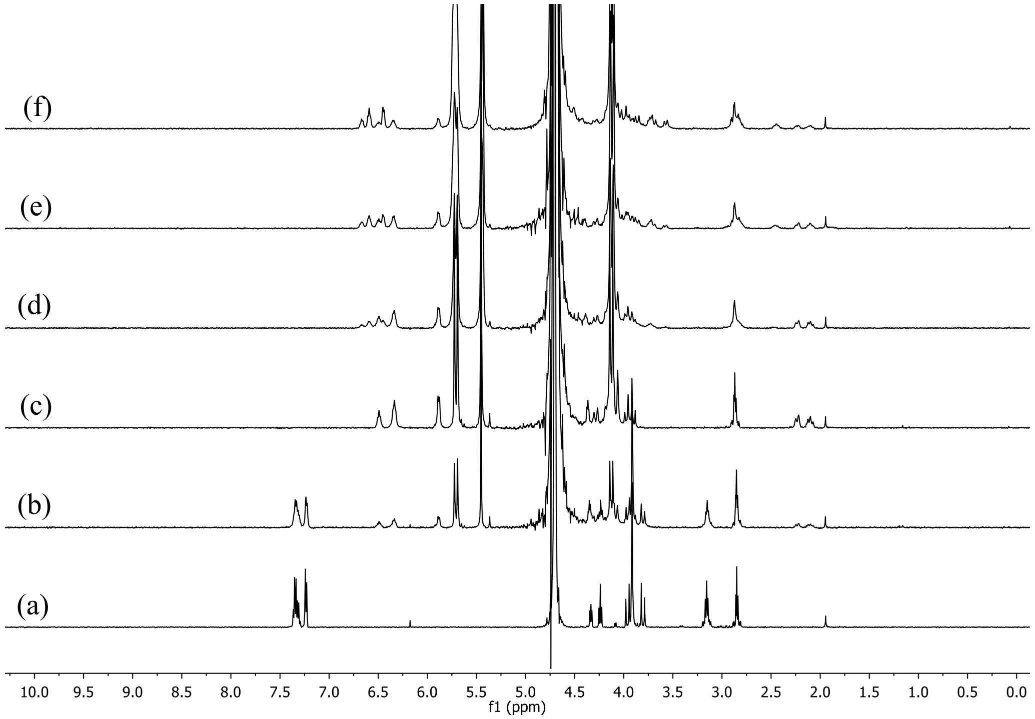

Figure S31. ^{1}H-NMR spectra of peptide FGGGC (1.0 mM) (a) in the absence of CB[8], and in the presence of (b) 0.25, (c) 0.50, (d) 0.75, (e) 1.0 and (f) 1.5 equiv CB[8] in D_2O.

9. Isothermal Titration Calorimetry (ITC) experiments

All ITC experiments were carried out in MilliQ water at 25 ºC on a Malvern MicroCal ITC200 instrument. The host (CB[7] or CB[8]) was located in the sample cell at concentrations ranging 30 – 100 µM and the titrant (Pt complex or peptide) was in the injection syringe at concentrations ranging from 0.3 to 1.5 mM (approximately 10 times higher). The titrations consisted of 20 injections with an injection spacing of 150 s. Raw data was analyzed (baseline correction, integration and fitting) with the Affinimeter software.

Table S2. Thermodynamic data obtained by ITC titrations of the species involved in this study.

System	K_1 [a]	ΔG_1 [b]	ΔH_1 [b]	$T\Delta S_1$ [b]	K_2 [a]	ΔG_2 [b]	ΔH_2 [b]	$T\Delta S_2$ [b]	β [c]	ΔG_β [b]	α [d]
**FGGGC vs CB[7]**	1.2 (±0.2) × 10^7	−9.66 (±0.09)	−13.80 (±0.02)	−4.15 (±0.09)							
**CB[8]^{Pt}·1$_2$ vs CB[7]**	6.3 (±0.2) × 10^6	−9.28 (±0.02)	−13.89 (±0.02)	−4.61 (±0.03)							
**2 vs CB[7]**	1.8 (±0.1) × 10^5	−7.18 (±0.01)	−8.47 (±0.02)	−1.28 (±0.03)							
**FGGGC vs CB[8]**	1.9 (±0.6) × 10^7	−9.9 (±0.2)	−11.4 (±0.1)	−1.5 (±0.2)	1.2 (±0.6) × 10^6	−8.3 (±0.3)	−10.4 (±0.1)	−2.0 (±0.3)	2.4 × 10^{13}	−18.2	0.26
**CB[8]^{Pt}·1$_2$ vs CB[8]**	2.4 (±0.3) × 10^6	−8.7 (±0.1)	−12.1 (±0.1)	−3.4 (±0.1)	1.7 (±0.4) × 10^6	−8.5 (±0.1)	−8.6 (±1.9)	−0.1 (±1.9)	4.0 × 10^{12}	−17.2	2.79
					$K_{Pt\text{-}Pt}$ [f]	$\Delta G_{Pt\text{-}Pt}$ [b]	$\Delta H_{Pt\text{-}Pt}$ [b]	$T\Delta S_{Pt\text{-}Pt}$ [b]			
**2 vs CB[8]** [e]					2.0 (±1.1) × 10^4	−5.9 (±0.3)	−6.7 (±0.9)	−0.9 (±1.0)	1.3 (±0.6) × 10^{13}	−17.9 (±0.3)	

[a] in M^{-1}. [b] in kcal/mol. [c] $\beta = K_1K_2$ [M^{-2}]. [d] Interaction parameter $\alpha = 4K_2/K_1$. [e] Binding constant corresponding to the equilibrium $\mathbf{2} + \mathbf{2} + \mathbf{CB[8]} \rightleftarrows \mathbf{CB[8]\cdot 2_2}$, in M^{-2}. [f] Dimerization constant of complex **2** [M^{-1}].

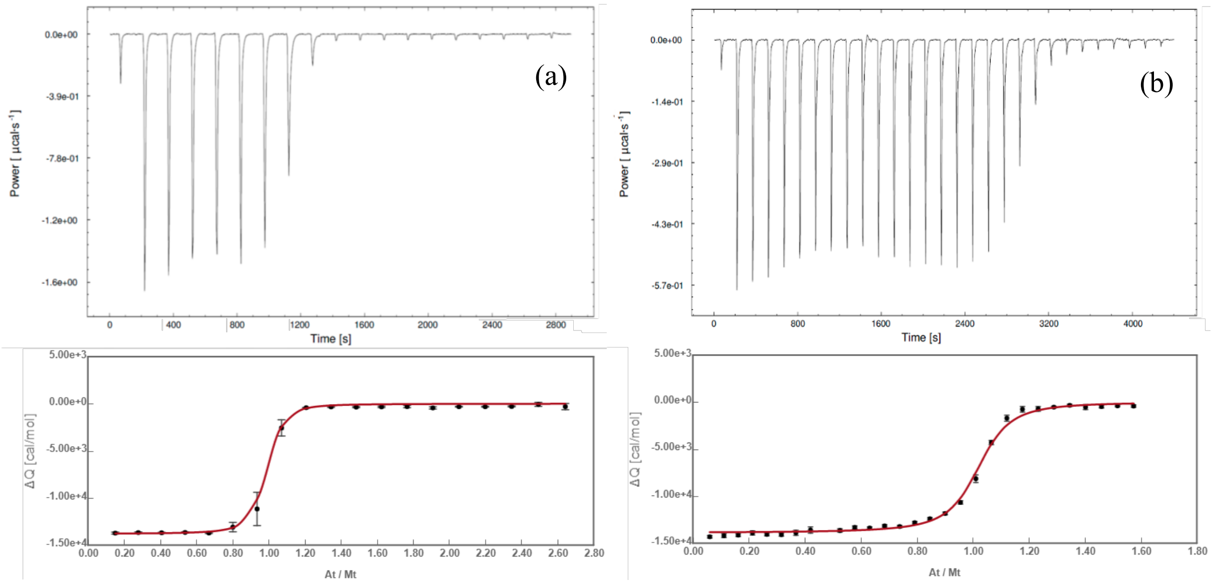

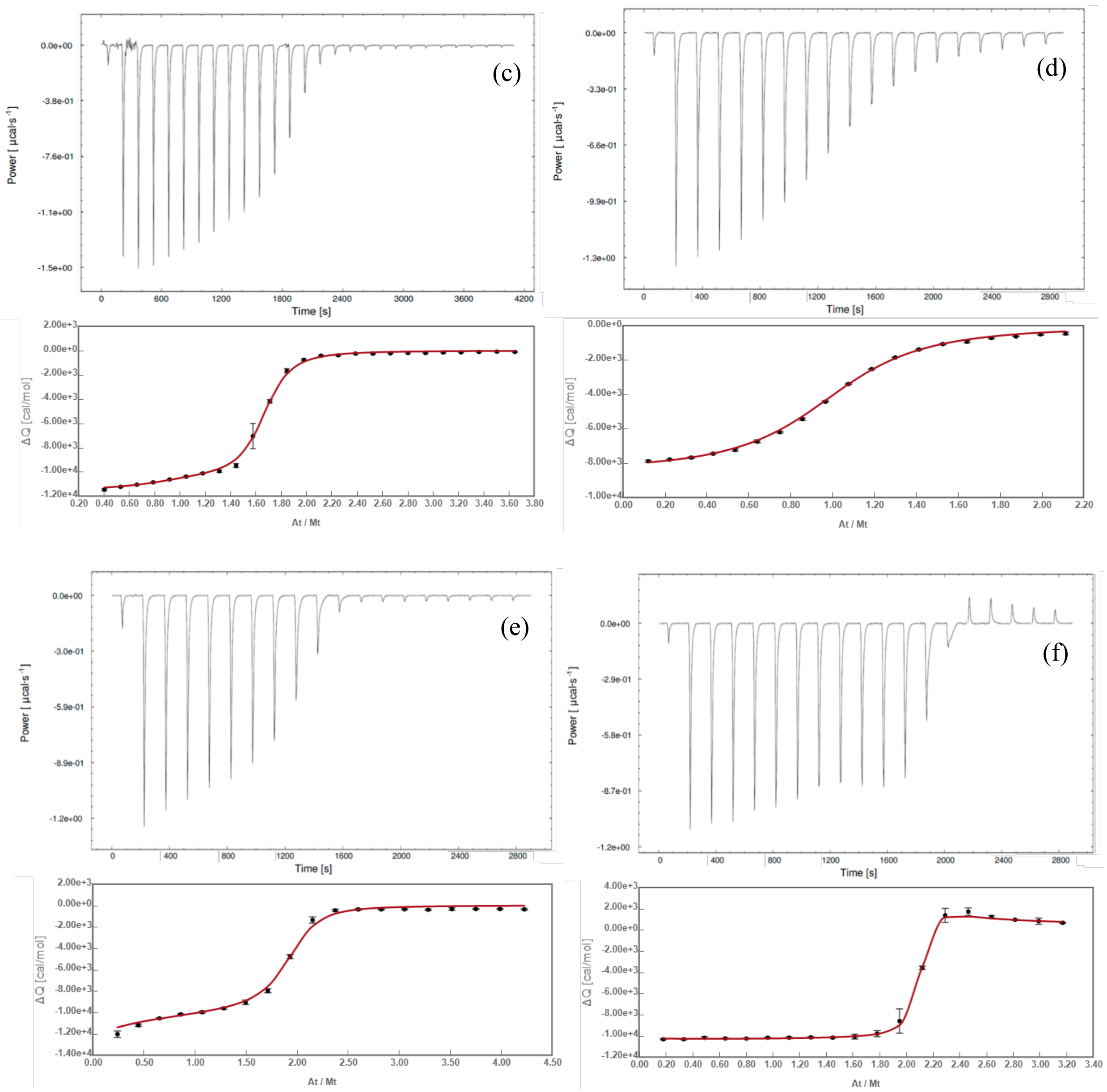

Figure S32. Representative enthalpograms for the titration of CB[7] (50 µM) with (a) FGGGC, (b) CB[8]^{Pt}·**1**$_2$, and (c) **2** (0.50 mM) in MilliQ water at 25 ºC. Representative enthalpograms for the titration of CB[8] (30 µM) with (d) FGGGC, (e) CB[8]^{Pt}·**1**$_2$, and (f) **2** (0.30 mM) in MilliQ water at 25 ºC.

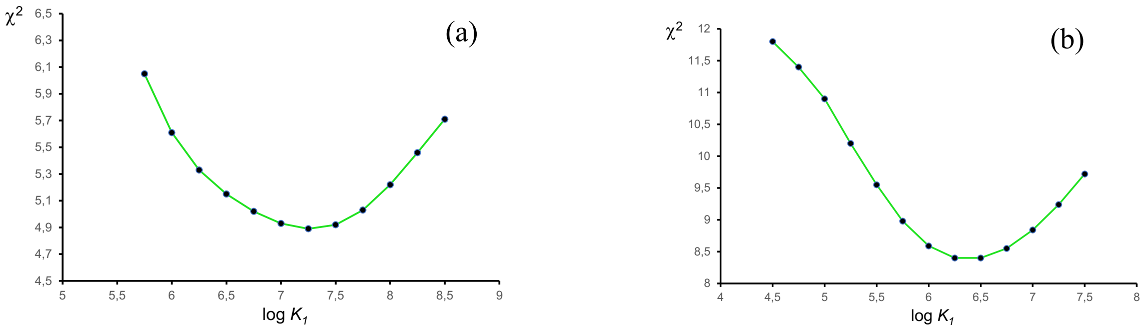

Figure S33. Goodness-of-fit value χ^2 as a function of binding constant K_1 as fixed variable while binding affinity K_2 is fitted, for the enthalpograms obtained upon titration of CB[8] with (a) FGGGC, and (b) CB[8]^{Pt}·$\mathbf{1}_2$ (see Figure S32, enthalpograms d and e).

10. Computational details

Conformational screening for assembly CB[8]^{Pt}·$\mathbf{1}_2$·CB[8]$^{Phe}{}_{HT}$ was carried out in Grimme's Conformer-Rotamer Ensemble Sampling Tool (CREST)[8,9] with the iMTD-GC algorithm and the generic GFN Force Field (GFN-FF)[10] with a surrogate structure that binds both peptides through a naphtho[1,2-b:8,7-b']dithiophene fragment (Figure S34). The distance between sulfur atoms was therefore controlled. The surrogate facilitated the computational load by removing Pt atoms and one CB[8] macrocycle. 11746 structures were generated, and the 37 best candidates were isolated for further evaluation (lowest energy within 25 kcal/mol of the most stable geometry). The surrogate fragment was then replaced by the CB[8]-secured platinum dimer in the 37 candidates, which were reoptimized with the semiempirical tight-binding method GFN2-xTB.[11,12] All GFN-FF and GFN2-xTB calculations were performed in conjunction with the GBSA solvation model.[13,14] The four structures with the lowest energy (within 10 kcal/mol of the most stable geometry) were finally reoptimized by density functional theory with the TURBOMOLE[15–18] suite of programs (version 7.2.1) at the B97-3c/def2-mTZVP level of theory with COSMO[19,20] solvation parameters. The m4 grid size was used and convergence criteria were 10^{-5} hartree. Enthalpic and entropic contributions at 25 ºC ($\Delta G_{T,\mathrm{xTB}}$) were obtained by vibrational analysis using GFN2-xTB-optimized structures. Free energies of solvation ($\Delta G_{\mathrm{solv,xTB}}$) were also calculated using the GBSA solvation model on GFN2-xTB-optimized structures. The relative stability (ΔG) of the four assemblies was calculated using equation (2), where $\Delta E_{\mathrm{B97\text{-}3c}}$ is the electronic contribution at 0 K calculated by DFT in the gas phase.

$$\Delta G = \Delta E_{\mathrm{B97-3c}} + \Delta G_{T,\mathrm{xTB}} + \Delta G_{\mathrm{solv,xTB}} \qquad (2)$$

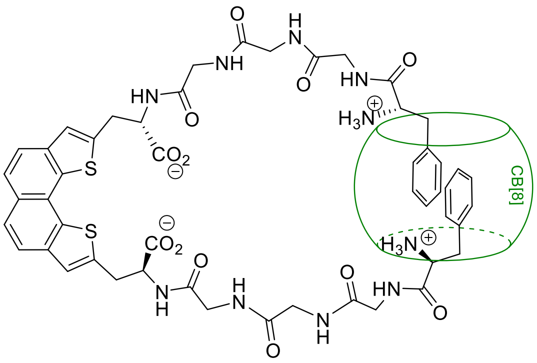

Figure S34. Surrogate used for initial conformational screening with the GFN-FF force field.

11. Coordinates of the most stable structure of pendant necklace $CB[8]^{Pt}\cdot 1_2\cdot CB[8]^{Phe}_{HT}$

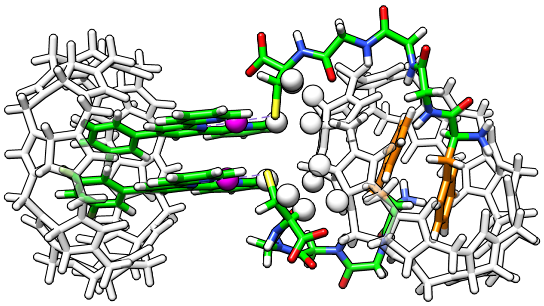

S	-0.4346860	8.1843884	2.9254170
C	1.0095628	8.6362825	3.9782137
C	-5.5448212	8.2227380	1.9958932
C	1.8923349	9.7095928	3.3569365
C	-6.4368726	8.7557780	0.8563795
N	2.6770384	9.2507243	2.2313585
H	1.2501362	10.4898751	2.9708638
C	-6.9506935	7.6180395	-0.0474223
N	-7.5617092	9.5166373	1.3731556
H	-5.8299250	9.4233326	0.2502369
C	-7.4069305	10.7257682	1.9160157
H	-8.4723388	9.1074242	1.2118074
C	2.5073602	9.7734869	1.0051574
H	3.5783640	8.8666812	2.4760698
C	-8.6687129	11.4499723	2.3550782
O	-6.2960548	11.2480659	2.0728335
O	1.5368752	10.4569212	0.6805699
C	3.6016882	9.4423091	-0.0111519
O	-8.1699658	7.3029202	0.0602920
O	-6.0944682	7.0932637	-0.7972429
C	2.8075748	10.3130849	4.4496973
O	3.9576137	9.8204763	4.5746650
O	2.2935321	11.2367728	5.1358275
N	3.6057390	10.3970987	-1.0904047
H	4.5750175	9.4138639	0.4730296
H	3.4183442	8.4542084	-0.4280174
H	-9.0161452	11.0061503	3.2909833
H	-9.4694206	11.3218864	1.6296423
N	-8.3684363	12.8539303	2.5252020
C	-9.3075795	13.8063040	2.5280734
H	-7.3867136	13.1038361	2.5100415
C	-8.8294773	15.2573890	2.5723157
O	-10.5189498	13.5684718	2.4432393
C	3.8643843	11.7028111	-0.8576403
H	3.0754704	10.1612654	-1.9116632
O	4.3351628	12.1120908	0.2007494
C	3.5702887	12.6238710	-2.0387597
H	-8.7591462	15.6043595	1.5425754
H	-9.6121573	15.8269460	3.0630244
N	-7.5636679	15.5180791	3.2155275
H	-6.7348227	15.5062411	2.6414346
C	-7.4332999	15.5904433	4.5476260
O	-8.3745889	15.4489208	5.3333923
C	-6.0282438	15.8210946	5.0938230
N	3.3673959	13.9870106	-1.6337337
H	2.6921233	12.2717244	-2.5781585
H	4.4132540	12.5891169	-2.7253758
H	2.5675341	14.1569459	-1.0377031
C	4.2751233	14.9535621	-1.8204078
O	5.3606753	14.7898187	-2.3874497
C	3.9244961	16.3337884	-1.2750722
H	-5.5987231	14.8493359	5.3266951
N	-5.0942338	16.5108551	4.2236739
H	-6.1424070	16.3617864	6.0289854
C	-5.1192696	17.8462761	4.1125066
H	-4.2795856	15.9787566	3.9450148
O	-5.9932852	18.5492534	4.6250747
C	-4.0225970	18.4997551	3.2509720
H	4.3818229	17.0603448	-1.9396003
H	4.3819046	16.4347202	-0.2960471
N	2.5129952	16.6254497	-1.1270728
H	2.1388339	16.6357486	-0.1893313
C	1.7605292	16.9669194	-2.1803248
C	0.2780055	17.2284985	-1.9090690
O	2.1906323	17.0359413	-3.3350753
C	-2.6075228	18.1396990	3.6641298
H	0.1417879	17.7611392	-0.9724836
C	-0.5078942	15.9105674	-1.8755716
N	-0.1954811	18.1153793	-3.0169352
H	0.0774700	15.1812986	-1.3216131
H	-0.6010794	15.5266410	-2.8907615
H	0.4220453	17.9380328	-3.8168339
H	-0.1402989	19.1245293	-2.8072386
H	-1.1573087	17.9410073	-3.3297440
C	-1.5207552	18.5582274	2.7099196
H	-5.2796964	16.6432893	0.4745886
C	-0.2048941	18.2355676	3.0394592
C	0.8437262	18.5974502	2.2147221
C	0.6054713	19.3104985	1.0477373
C	-0.6984322	19.6267723	0.7059266
C	-1.7521685	19.2425099	1.5227642
H	1.4338978	19.6288072	0.4302084
H	-0.9030499	20.1950340	-0.1872278
H	-2.7544023	19.4919750	1.2058313
C	-4.3140944	16.5102638	0.0095898
C	-3.2218449	16.0811229	0.7501265
C	-1.9935104	15.8872744	0.1428082
C	-1.8491329	16.0743786	-1.2282388
C	-2.9600635	16.4595444	-1.9718114
C	-4.1777292	16.7013535	-1.3558791
H	-3.3300790	15.8641374	1.8021171
H	-1.1453614	15.5724097	0.7363679
H	-2.8789860	16.5374853	-3.0450777
H	-5.0243453	17.0100898	-1.9496094
S	-3.7384708	8.4008188	1.7200888
H	1.8522499	18.3354051	2.4826389
H	-0.0051071	17.7017163	3.9581662
N	-4.2491711	19.9742121	3.3234439
H	-4.1968010	18.1904532	2.2223471
H	-2.5411606	17.0625518	3.8083736
H	-2.4079158	18.5651257	4.6490729
H	-4.5525875	20.3982510	2.4335398
H	-3.4150791	20.5052792	3.6238255
H	-5.0016000	20.1305751	4.0053922
N	-6.8273968	14.3090679	-2.0690120
C	-6.5301038	13.9880518	-3.4496369
C	-6.7350861	15.3458121	-4.1654930
N	-7.3562435	16.1625260	-3.1590703
C	-7.3852766	15.5552144	-1.9242954
H	-7.1906542	13.1998785	-3.8088808
H	-7.3670602	15.2829563	-5.0502054
N	-5.1587937	13.6621029	-3.7371124
N	-5.3808961	15.7183067	-4.5381502
C	-4.4975557	14.6725724	-4.3873494
O	-7.8509154	16.0251340	-0.8977453
O	-3.3441845	14.6401300	-4.7881247
N	-0.7013063	13.7535496	4.1218532
C	-0.0291021	12.7975738	3.2753031
C	-1.1585801	12.2091111	2.4062811
N	-2.3163625	12.9696947	2.8200077
C	-2.0387696	13.8488490	3.8301211
H	0.5048073	12.0770950	3.8943445
H	-1.3221365	11.1391714	2.5532249
N	0.8604228	13.3909719	2.3038162
N	-0.7375670	12.4783658	1.0556587
C	0.4523206	13.1667632	1.0142373
O	-2.8510699	14.5828500	4.3941556
O	1.0302890	13.5230954	-0.0046119
N	2.0244164	16.8439268	6.1461087
C	2.9852503	16.1402336	5.3389746
C	2.2133430	14.8787716	4.8806755
N	0.8603001	15.1393691	5.3322873
C	0.7898860	16.2433949	6.1508592
H	3.8804312	15.9195748	5.9188505
H	2.5889650	13.9495247	5.3075651
N	3.3452082	16.7798311	4.0886972
N	2.3832749	14.8802186	3.4487056
C	3.0487879	15.9912182	3.0007062
O	-0.1900316	16.6024673	6.7863225
O	3.3467892	16.2286413	1.8361624
N	1.4583408	21.1090144	5.0921528
C	2.8870154	21.1935557	4.8545983
C	3.3930871	19.8115965	5.3348273
N	2.2549771	19.2541358	6.0124984
C	1.1228069	20.0178940	5.8614944
H	3.3158161	22.0315976	5.4029753
H	4.2475023	19.8671065	6.0080399
N	3.2889684	21.2357952	3.4751506
N	3.7654405	19.1628915	4.0939305
C	3.7750810	20.0307352	3.0279261
O	0.0277233	19.7918192	6.3511303
O	4.1694730	19.7845440	1.8982551
C	-0.1468331	14.1148639	5.4005898
H	0.2761401	13.2169698	5.8551470
H	-0.9471372	14.4913684	6.0277044
C	2.2266779	13.7032707	2.6374206
H	2.7725773	13.8460524	1.7121393
H	2.6532679	12.8593488	3.1785853
N	-1.4025204	22.8109067	2.0363787
C	-0.2197862	23.4998132	1.5641108
C	0.8106099	23.2714334	2.6926835
N	0.0805817	22.4766391	3.6521744
C	-1.2115677	22.2518607	3.2761408
H	-0.4445584	24.5513422	1.3870832
H	1.1609359	24.1913660	3.1594348
N	0.4082246	22.9104300	0.4088168
N	1.8976588	22.6000773	2.0272435
C	1.6816734	22.4550939	0.6800944
O	-2.0700414	21.6867457	3.9540838
O	2.4763055	22.0278651	-0.1431427
N	-4.5288817	21.9041245	-1.2539406
C	-3.4665623	22.4814264	-2.0289852
C	-2.5262868	23.0791649	-0.9670471
N	-3.0369445	22.5197213	0.2746842
C	-4.2421739	21.8860146	0.0814449
H	-3.8533287	23.2094886	-2.7390293
H	-2.5336567	24.1667575	-0.9312322
N	-2.6215702	21.5071849	-2.7003984
N	-1.2302448	22.5861425	-1.3448358
C	-1.3084117	21.6266252	-2.3160789
O	-4.9556321	21.4023168	0.9615197
O	-0.3601870	20.9837667	-2.7708858
C	-2.7091251	23.1360593	1.5406941
H	-2.7831052	24.2199659	1.4275913
H	-3.4431409	22.8188124	2.2719913
C	-0.0035795	23.2205739	-0.9308522
H	-0.1380539	24.2985711	-1.0329935
H	0.7866740	22.8977141	-1.5988904
C	3.1889856	22.3976461	2.6375701
H	3.9122526	22.2949613	1.8360374
H	3.4375733	23.2751270	3.2325087
C	-5.8258296	21.6054384	-1.8062541
H	-6.1093777	22.4349346	-2.4552171
H	-6.5339615	21.5373492	-0.9883000
C	-2.9819904	20.9947725	-4.0026119
H	-2.0870757	20.5945579	-4.4653625
H	-3.3504830	21.8220837	-4.6120517
C	2.2986628	18.0749388	6.8373167
H	3.2835077	18.0125050	7.2948351
H	1.5519436	18.1791634	7.6170017
C	4.3226528	17.8423911	4.0188744
H	4.8371680	17.7637709	3.0669478
H	5.0414304	17.7030379	4.8266033
C	0.5745570	22.2346307	4.9837714
H	1.0868864	23.1348317	5.3252299
H	-0.2773865	22.0452401	5.6274484
N	-3.9766850	19.9606745	-3.9771322
C	-5.3915853	20.2467752	-3.8862491
C	-6.0581918	18.9495865	-4.4014736
N	-4.9304501	18.0896644	-4.6897519
C	-3.7310140	18.7041935	-4.4681043
H	-5.6380463	21.1276531	-4.4778463
H	-6.6597003	19.0905663	-5.2979830
N	-5.8944560	20.3737230	-2.5409128
N	-6.8845406	18.5348082	-3.2970481
C	-6.8305657	19.4080165	-2.2360358
O	-2.6162044	18.2291784	-4.6879052
O	-7.5056013	19.3596735	-1.2216969
C	-7.8682641	17.4841836	-3.3915791
H	-8.6295830	17.6832729	-2.6453922
H	-8.3212062	17.5134276	-4.3806753
C	-5.0721844	16.8353280	-5.3879643
H	-4.1325412	16.6199475	-5.8850018
H	-5.8533860	16.9504141	-6.1392038
C	-2.6169606	12.2389587	-1.9311026
N	-3.9760975	12.2435537	-2.1644934
C	-4.7454109	11.9544010	-0.9819828
C	-3.6711510	11.7689080	0.1113844
N	-2.4357447	11.9942252	-0.5921869
H	-5.3662490	11.0715620	-1.1469077
H	-3.6835015	10.7771303	0.5720696
N	-4.0006225	12.7679361	1.1022466
N	-5.5546549	13.0287775	-0.4683134
C	-5.1597779	13.4258558	0.7815193
O	-5.7525363	14.2184685	1.5024821
O	-1.7365665	12.3633531	-2.7672698
C	-4.5550787	12.3847869	-3.4740409
H	-5.3126430	11.6148696	-3.6139829
H	-3.7577890	12.2374496	-4.1952662
C	-6.8507360	13.3429504	-1.0099125
H	-7.4583330	13.7559623	-0.2119220
H	-7.3089844	12.4200585	-1.3680045
C	-1.1607667	11.6445230	-0.0355929
H	-1.1986371	10.6172528	0.3314684
H	-0.4224334	11.7191816	-0.8248087
C	-3.6504765	12.5598540	2.4841507
H	-3.7645830	11.4996803	2.7153677
H	-4.3406103	13.1265430	3.0965689
H	1.5954008	7.7507254	4.2115584
H	0.6046208	9.0264372	4.9098416
H	-5.7489124	8.8018168	2.8913546
H	-5.7762588	7.1839481	2.2189100
H	-2.2323162	5.7412721	-4.2601502
C	-2.5158720	6.5096028	-3.5582641
C	-3.2276079	8.4054200	-1.7218863
C	-2.6247092	6.1999921	-2.2133369
C	-2.7642590	7.8037634	-3.9836694
C	-3.1223054	8.7632243	-3.0515068

N	-2.9752223	7.1627304	-1.3028623
H	-2.6776691	8.0594631	-5.0289876
H	-3.3181269	9.7802972	-3.3501342
H	-3.5167080	9.0924639	-0.9443757
C	-2.3988909	4.8645570	-1.6625762
C	-2.1767069	2.4920827	-0.2718628
C	-2.0539124	3.7172380	-2.3466445
N	-2.5940998	4.8029781	-0.3352601
C	-2.5021813	3.6677182	0.3780892
C	-1.9442136	2.5086710	-1.6523930
H	-1.9217763	3.7503476	-3.4137460
H	-2.1130802	1.5725220	0.2853315
C	-2.7564182	3.8727825	1.8055223
C	-3.2007512	4.4859155	4.4457934
C	-2.6501115	2.8803812	2.7623342
N	-3.0828605	5.1571391	2.1711221
C	-3.2881659	5.4500878	3.4580787
C	-2.8802729	3.1863923	4.0949538
H	-2.4029586	1.8725653	2.4674841
H	-3.5042860	6.4855730	3.6701647
H	-2.8018127	2.4173117	4.8486898
H	-3.3800036	4.7644573	5.4726783
C	-1.5915121	1.2813450	-2.3721219
C	-0.8972123	-1.0637834	-3.7459805
C	-0.8200884	1.3498832	-3.5338885
C	-2.0089242	0.0387248	-1.8947041
C	-1.6510269	-1.0965343	-2.5884963
C	-0.4874148	0.1809475	-4.1833759
H	-0.4514379	2.2876277	-3.9189081
H	-2.6167651	-0.0499142	-1.0097983
F	-2.0390674	-2.2967028	-2.1114458
F	0.2974549	0.2504899	-5.2767824
H	-0.6304898	-1.9640619	-4.2758304
Pt	-3.1118674	6.4565349	0.6050240
N	5.7025343	1.0124193	-5.3433493
C	5.6166601	2.4461139	-5.2203566
C	4.5977674	2.8426823	-6.3180557
N	4.2312102	1.5803967	-6.9088399
C	4.9271050	0.5231104	-6.3684387
H	6.5990491	2.8998560	-5.3505532
H	5.0071280	3.5137772	-7.0730397
N	5.0017854	2.9270906	-4.0052388
N	3.5468546	3.5025167	-5.5794675
C	3.8109405	3.5671452	-4.2364043
O	4.8943260	-0.6348709	-6.7585578
O	3.1192558	4.1234412	-3.3909972
N	3.5806880	-3.5966021	1.5775869
C	4.1897723	-2.6609935	2.4892160
C	5.2687287	-1.9610688	1.6305742
N	5.1002954	-2.5549872	0.3239071
C	4.1676511	-3.5709807	0.3329532
H	4.5942080	-3.1861294	3.3548657
H	6.2847667	-2.1244996	1.9908842
N	3.3464577	-1.5708284	2.9150487
N	4.9234942	-0.5647451	1.7268844
C	3.8320521	-0.3455964	2.5298643
O	3.9282661	-4.3336880	-0.5919384
O	3.3893680	0.7436388	2.8708454
N	-0.8224128	-4.6775540	2.1165994
C	-0.6563515	-3.8218091	3.2591303
C	0.8789517	-3.7180811	3.4169298
N	1.3757970	-4.3825342	2.2312683
C	0.3745156	-5.0122150	1.5283132
H	-1.1580911	-4.2436389	4.1298089
H	1.2619721	-4.2022568	4.3149659
N	-1.0630837	-2.4441584	3.0634475
N	1.1122903	-2.2992441	3.4767856
C	-0.0246453	-1.5643201	3.2579905
O	0.5225833	-5.7685962	0.5793950
O	-0.1055211	-0.3412386	3.2622563
N	-4.4598412	-3.9142159	-0.5451025
C	-4.8675632	-3.1076135	0.5761822
C	-3.8487003	-3.4857183	1.6798682
N	-2.9814532	-4.4374747	1.0291828
C	-3.3870316	-4.7231104	-0.2551851
H	-5.9022439	-3.3211246	0.8439686
H	-4.3061745	-3.9292687	2.5641010
N	-4.6777796	-1.6835268	0.4165839
N	-3.2336585	-2.2240849	2.0136231
C	-3.7460788	-1.1798975	1.2896552
O	-2.9061291	-5.5740554	-0.9893985
O	-3.4480089	0.0023433	1.4170213
C	2.7664207	-4.6958404	2.0335877
H	3.1854068	-5.0665372	2.9707250
H	2.8172498	-5.4809133	1.2869483
C	2.3443422	-1.7177411	3.9332320
H	2.1285529	-0.7283625	4.3212973
H	2.7371780	-2.3386433	4.7386596
N	-4.7926251	-1.9304471	-4.5821686
C	-5.8470514	-1.1510950	-3.9617071
C	-5.9810933	-1.7964645	-2.5657959
N	-5.1955068	-2.9942898	-2.6713848
C	-4.5170669	-3.0755081	-3.8630086
H	-6.7589082	-1.2058079	-4.5570509
H	-7.0082753	-2.0385738	-2.2919598
N	-5.5176456	0.2168111	-3.6731886
N	-5.4538586	-0.7725417	-1.6849354
C	-5.3078128	0.4374180	-2.3324926
O	-3.8230473	-4.0100530	-4.2359035
O	-5.0644838	1.5128007	-1.8067505
N	-2.2451968	0.2939595	-7.6839600
C	-3.2846417	1.2780964	-7.5268054
C	-4.3586740	0.5474868	-6.6875432
N	-3.7594616	-0.7392999	-6.4193119
C	-2.5586399	-0.9007442	-7.0779973
H	-3.6432640	1.6081322	-8.5020711
H	-5.3069297	0.4205036	-7.2105026
N	-2.9432759	2.4170286	-6.7082417
N	-4.5325421	1.4034646	-5.5401782
C	-3.7477337	2.5281183	-5.6003834
O	-1.9065360	-1.9325243	-7.1392367
O	-3.7804896	3.4769913	-4.8278854
C	-4.5480034	-1.8682657	-6.0002969
H	-5.5042518	-1.8426194	-6.5288870
H	-4.0137213	-2.7701151	-6.2786341
C	-5.6431233	1.2782448	-4.6339824
H	-6.5550169	1.1169256	-5.2100992
H	-5.7243039	2.2127480	-4.0895786
C	-5.6277684	-0.8391141	-0.2580252
H	-5.5133083	0.1678157	0.1298066
H	-6.6335078	-1.1989625	-0.0316165
C	-1.2164053	0.4121632	-8.6860307
H	-1.6556834	0.8668489	-9.5755927
H	-0.8717043	-0.5866051	-8.9308756
C	-2.1444566	3.5080507	-7.1939393
H	-2.3377595	4.3613291	-6.5533222
H	-2.4477384	3.7520717	-8.2125052
C	-2.0753063	-5.2866479	1.7608421
H	-2.5682994	-5.6219360	2.6742286
H	-1.8588440	-6.1467812	1.1367702
C	-2.4352584	-2.0278912	3.1912748
H	-2.4354195	-0.9671985	3.4178970
H	-2.8949943	-2.5713979	4.0185471
C	-5.2654971	-4.0770498	-1.7263951
H	-6.3064129	-4.2026811	-1.4273422
H	-4.9281057	-4.9776410	-2.2275231
N	-0.7287941	3.2567256	-7.1939398
C	-0.0408427	2.6211074	-8.2892430
C	1.4466707	2.9461161	-8.0176019
N	1.4125109	3.5879622	-6.7195385
C	0.1302583	3.8171185	-6.2829017
H	-0.4083531	3.0079712	-9.2394113
H	1.8910385	3.6131126	-8.7563338
N	-0.0656652	1.1760395	-8.2866630
N	2.0884217	1.6594681	-8.0490601
C	1.1945802	0.6292942	-8.2259006
O	-0.1916194	4.4349470	-5.2726481
O	1.4764633	-0.5534350	-8.3515103
C	3.5129066	1.4874891	-8.1537182
H	3.6973804	0.5004431	-8.5634892
H	3.9024528	2.2393293	-8.8411854
C	2.5395809	4.3167489	-6.2025426
H	2.1675574	5.0129039	-5.4588464
H	3.0014658	4.8780470	-7.0166078
C	5.3576025	1.8501203	-0.6695581
N	5.7865837	1.8668542	-1.9808212
C	6.7817684	0.8439921	-2.2371152
C	6.7034746	-0.0379212	-0.9724857
N	5.8849585	0.7288193	-0.0764428
H	7.7600318	1.2993711	-2.3930067
H	7.6749651	-0.2416037	-0.5213511
N	6.1092259	-1.2665917	-1.4657031
N	6.4777538	-0.0681457	-3.3042381
C	6.1105898	-1.3106078	-2.8460492
O	5.8631330	-2.2898914	-3.5343411
O	4.6607333	2.6945133	-0.1275773
C	5.7195598	3.0692626	-2.7679077
H	6.7315563	3.4127085	-2.9913056
H	5.2109433	3.8197458	-2.1719533
C	6.7198973	0.2277953	-4.6921382
H	6.7920661	-0.7146803	-5.2237995
H	7.6713507	0.7547818	-4.7677861
C	5.8228513	0.4873433	1.3377331
H	6.8259712	0.2460357	1.6913218
H	5.4842827	1.3989181	1.8179276
C	6.1279659	-2.4769979	-0.6826319
H	7.1014342	-2.5741021	-0.1956247
H	5.9894480	-3.3083068	-1.3652430
H	1.0887300	5.2673188	-2.9262981
C	0.8131378	6.0576194	-2.2468968
C	0.1339595	8.0408975	-0.4872071
C	0.6654648	5.7927687	-0.8977150
C	0.6201918	7.3479370	-2.7172286
C	0.2791974	8.3508986	-1.8262274
N	0.3217386	6.8012535	-0.0285131
H	0.7371405	7.5627542	-3.7687543
H	0.1211173	9.3657162	-2.1573561
H	-0.1104135	8.7726502	0.2628332
C	0.8581102	4.4782948	-0.2857013
C	1.1413599	2.1947636	1.2442472
C	1.1965562	3.2899984	-0.9044837
N	0.6684401	4.4885316	1.0460223
C	0.8171536	3.4040859	1.8271262
C	1.3331484	2.1262924	-0.1396171
H	1.3539925	3.2685394	-1.9693292
H	1.2820808	1.3235394	1.8607768
C	0.5924361	3.6838069	3.2450426
C	0.1060823	4.4015156	5.8500067
C	0.7071784	2.7355807	4.2458631
N	0.2440682	4.9788400	3.5468303
C	-0.0071124	5.3135299	4.8174726
C	0.4669654	3.0965812	5.5618920
H	0.9668632	1.7206634	3.9902785
H	-0.3136363	6.3342656	4.9800313
H	0.5534805	2.3636890	6.3501860
H	-0.0977280	4.7210148	6.8603139
C	1.6503012	0.8462391	-0.7795572
C	2.1777428	-1.5991283	-2.0461399
C	2.3880655	0.8142823	-1.9629835
C	1.1755067	-0.3453343	-0.2288025
C	1.4431882	-1.5311821	-0.8780336
C	2.6254665	-0.4005042	-2.5674151
H	2.7831862	1.7141711	-2.4043792
H	0.5664419	-0.3561275	0.6615716
F	0.9338849	-2.6723415	-0.3732917
F	3.3057371	-0.4219906	-3.7308701
H	2.3759087	-2.5395700	-2.5345253
Pt	0.1695611	6.1896293	1.9067695

12. References

1 F. Diederich, P. J. Stang and R. R. Tykwinski, in *Modern supramolecular chemistry*, Wiley-VCH Verlag GmbH & Co. KGaA, 2008.

2 W. L. F. Armarego and C. L. L. Chai, *Purification of Laboratory Chemicals*, Butterworth-Heinemann, 2012.

3 K. Kotturi and E. Masson, *Chem. - Eur. J.*, 2018, **24**, 8670–8678.

4 E. O. Stejskal and J. E. Tanner, *J. Chem. Phys.*, 1965, **42**, 288–292.

5 D. H. Wu, A. Chen and C. S. Johnson, *J. Magn. Reson. Ser. A*, 1995, **115**, 123–126.

6 C. S. Johnson, *Prog. Nucl. Magn. Reson. Spectrosc.*, 1999, **34**, 203–256.

7 P. W. Kuchel, G. Pagès, K. Nagashima, S. Velan, V. Vijayaragavan, V. Nagarajan and K. H. Chuang, *Concepts Magn. Reson. Part A*, 2012, 205–214.

8 S. Grimme, *J. Chem. Theory Comput.*, 2019, **15**, 2847–2862.

9 P. Pracht, F. Bohle and S. Grimme, *Phys. Chem. Chem. Phys.*, 2020, **22**, 7169–7192.

10 S. Spicher and S. Grimme, *Angew. Chem., Int. Ed.*, 2020, **59**, 15665–15673.

11 S. Grimme, C. Bannwarth and P. Shushkov, *J. Chem. Theory Comput.*, 2017, **13**, 1989–2009.

12 C. Bannwarth, S. Ehlert and S. Grimme, *J. Chem. Theory Comput.*, 2019, **15**, 1652–1671.

13 M. Brieg, J. Setzler, S. Albert and W. Wenzel, *Phys. Chem. Chem. Phys.*, 2017, **19**, 1677–1685.

14 A. V. Onufriev and D. A. Case, *Annu. Rev. Biophys.*, 2019, **48**, 275–296.

15 *TURBOMOLE V7.2.1 2015, a development of University of Karlsruhe and Forschungszentrum Karlsruhe GmbH, 1989-2007, TURBOMOLE GmbH, since 2007; available from http://www.turbomole.com*

16 R. Ahlrichs, M. Bär, M. Häser, H. Horn and C. Kölmel, *Chem. Phys. Lett.*, 1989, **162**, 165–169.

17 M. Sierka, A. Hogekamp and R. Ahlrichs, *J. Chem. Phys.*, 2003, **118**, 9136–9148.

18 P. Deglmann, K. May, F. Furche and R. Ahlrichs, *Chem. Phys. Lett.*, 2004, **384**, 103–107.

19 A. Klamt and G. Schüürmann, *J. Chem. Soc. Perkin Trans. 2*, 1993, 799–805.

20 A. Klamt, *J. Phys. Chem.*, 1995, **99**, 2224–2235.
